# Imperfect centered miRNA binding sites are common and can mediate repression of target mRNAs

**DOI:** 10.1186/gb-2014-15-3-r51

**Published:** 2014-03-14

**Authors:** Hilary C Martin, Shivangi Wani, Anita L Steptoe, Keerthana Krishnan, Katia Nones, Ehsan Nourbakhsh, Alexander Vlassov, Sean M Grimmond, Nicole Cloonan

**Affiliations:** 1 Queensland Centre for Medical Genomics, Institute for Molecular Bioscience, The University of Queensland, Brisbane, St Lucia QLD 4072, Australia; 2 Wellcome Trust Centre for Human Genetics, University of Oxford, Oxford OX3 7BN, UK; 3 Life Technologies, 2130 Woodward St, Austin, TX 78744, USA; 4 Wolfson Wohl Cancer Research Centre, Institute of Cancer Sciences, University of Glasgow, Glasgow G61 1BD, UK; 5 QIMR Berghofer Medical Research Institute, Genomic Biology Laboratory, 300 Herston Road, Herston, QLD 4006, Australia

## Abstract

**Background:**

MicroRNAs (miRNAs) bind to mRNAs and target them for translational inhibition or transcriptional degradation. It is thought that most miRNA-mRNA interactions involve the seed region at the 5′ end of the miRNA. The importance of seed sites is supported by experimental evidence, although there is growing interest in interactions mediated by the central region of the miRNA, termed centered sites. To investigate the prevalence of these interactions, we apply a biotin pull-down method to determine the direct targets of ten human miRNAs, including four isomiRs that share centered sites, but not seeds, with their canonical partner miRNAs.

**Results:**

We confirm that miRNAs and their isomiRs can interact with hundreds of mRNAs, and that imperfect centered sites are common mediators of miRNA-mRNA interactions. We experimentally demonstrate that these sites can repress mRNA activity, typically through translational repression, and are enriched in regions of the transcriptome bound by AGO. Finally, we show that the identification of imperfect centered sites is unlikely to be an artifact of our protocol caused by the biotinylation of the miRNA. However, the fact that there was a slight bias against seed sites in our protocol may have inflated the apparent prevalence of centered site-mediated interactions.

**Conclusions:**

Our results suggest that centered site-mediated interactions are much more frequent than previously thought. This may explain the evolutionary conservation of the central region of miRNAs, and has significant implications for decoding miRNA-regulated genetic networks, and for predicting the functional effect of variants that do not alter protein sequence.

## Background

MicroRNAs (miRNAs) are small non-coding RNAs (approximately 22 nucleotides) that regulate the expression of protein-coding genes in association with the RNA-induced silencing complex (RISC). They are present in most eukaryotic cells, have been predicted to affect the expression of over 60% of mammalian genes [1], and have been implicated in multiple cellular processes and human diseases. They can repress their targets via several mechanisms: preventing translation initiation or elongation, directing mRNA cleavage, and prompting mRNA decay [2]. It was thought that miRNA-mRNA interactions almost always involved contiguous and perfect Watson-Crick base-pairing between the miRNA 'seed region' and the mRNA target [3], and that binding outside the seed served to augment imperfect seed binding [3].

The attractive simplicity of using nucleotide complementarity to identify mRNA targets has given rise to many bioinformatics tools. These are based (to differing extents) on complementarity to the seed, evolutionary conservation, and free energy of binding [4]. The presence of canonical seed sites (in the absence of other more extensive complementarity to the miRNA) is by far the most discriminatory feature for target prediction [5], and incorporation of other modes of binding dramatically reduces the accuracy of these programs [6,7]. However, results from our lab [8] and others [6,9,10] suggest that even the best algorithms have false positive rates of 20 to 40%, and any non-seed interactions will be absent from these predictions. In order to comprehensively understand the cohort of direct miRNA targets, unbiased transcriptome-wide experimental methods are needed.

**Figure 1:**
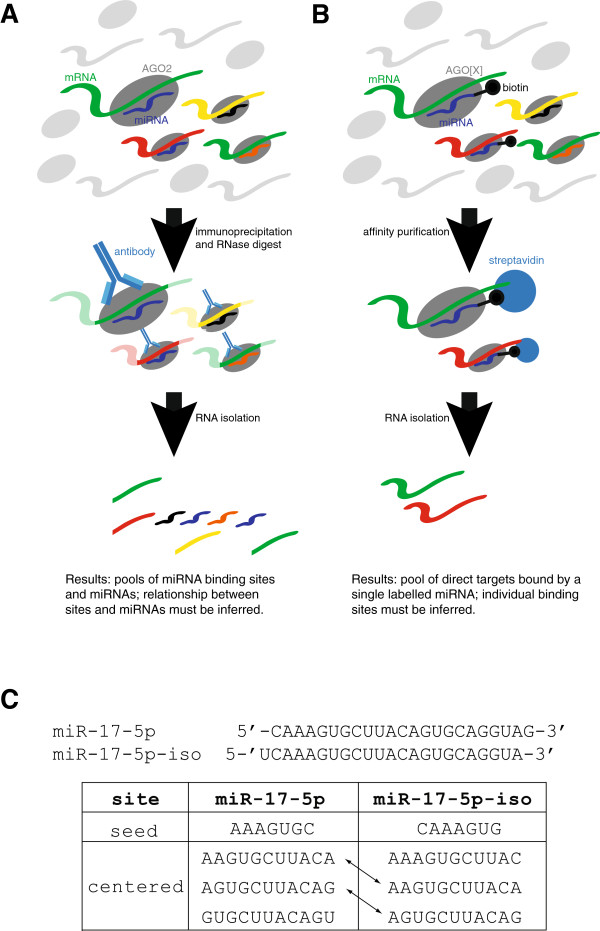
**Methods for ascertaining miRNA targets. (A)** In the HITS-CLIP method [11], RISC is pulled down using an antibody against AGO2. RNAse digestion then reduces the full length mRNA targets to short fragments that can be sequenced along with the miRNAs using high-throughput sequencing. This method uncovers RISC occupancy on individual transcripts and therefore captures the binding sites, but it cannot directly link them to a particular miRNA. This information must be inferred from the pool of miRNAs present. **(B)** In this study we employed a biotin pull-down method [13], in which a synthetic biotinylated miRNA is transfected into cells, integrates into RISC, and is then pulled down using streptavidin beads. The associated mRNAs are then profiled by microarray or RNAseq. This method does not reveal the exact miRNA binding sites, but it does preserve the relationship between the miRNA and its targets, allowing the exploration of closely related miRNA species. **(C)** We examined four shifted isomiRs and their canonical partners, which have different seed sites but share centered sites (defined as 11 bp starting at position 3, 4 or 5). miR-17-5p and its isomiR are illustrated here.

A common experimental approach to determining miRNA targets has been to transfect cells with an exogenous miRNA or knock down an endogenous one and measure the resulting change in mRNA or protein levels of each gene [6,7]. This approach is problematic because many of the effects observed may be due to changes in the levels of transcription factors that are targeted by the miRNA, rather than being direct consequences of miRNA binding [8]. Sequencing-based methods such as high-throughput sequencing of RNAs isolated by crosslinking immunoprecipitation (HITS-CLIP) and photoactivatable-ribonucleoside-enhanced crosslinking and immunoprecipitation (PAR-CLIP) [11,12] identify fragments of mRNA bound by Argonaute proteins (usually AGO2) and a pool of miRNAs, and infer the interactions between them bioinformatically (Figure 1A). This approach is an excellent tool to understand the genome-wide occupancy of RISCs, but is not suitable for the comparative evaluation of sites between closely related miRNAs, since it does not retain the information about which miRNAs are bound to which sites. An alternative approach is to transfect cells with a synthetic biotinylated miRNA, perform streptavidin purification, and profile the captured mRNA population via microarray or sequencing (Figure 1B) [13-18]. Although the introduction of a synthetic molecule into a cellular system is not ideal, this approach provides high-quality data for the inference of binding sites for single miRNAs, and provides the opportunity to measure target affinity between very closely related miRNAs, even those with a single nucleotide difference [13].

While most experimental effort has been directed at verifying seed-based interactions, non-seed based interactions have also been demonstrated, albeit far less frequently [3,7,19,20]. The most common variations appear to be 'seed-like' with mismatches or wobble in positions 5, 6, and 7 [20], and 'G-bulge' sites, in which the mRNA nucleotide that would normally pair with position 6 of the miRNA is bulged out of the interaction [19]. The strong evolutionary conservation of the central region of miRNAs [7] prompted a successful search for 'centered site'-mediated miRNA activity, demonstrating that 11 nucleotides of perfect complementarity starting at position 3, 4 or 5 could inhibit mRNA translation [21]. However, these centered sites were found only occasionally within the human transcriptome, similar in frequency to the 3′ supplementary and 3′ complementary sites that together account for <10% of interactions [3]. It remains an open question as to why the central region of miRNAs is strongly conserved; it may be driven by miRNA-mRNA interactions and/or by miRNA biogenesis. We hypothesized that imperfect centered sites could mediate miRNA-mRNA interactions, and that they are more commonly used than results from degradome sequencing would suggest. To test this hypothesis, we employed the biotin pull-down approach [13] to identify the direct miRNA targets for 10 carefully selected miRNAs (Table S1 in Additional file 1). We included two closely related miRNAs, miR-10a and miR-10b, which share identical seed sites, but differ by a single nucleotide in the center region. We also included four shifted isomiRs, which are miRNAs produced from the same pre-miRNA as the 'canonical' mature miRNA, but differing in 5′ and/or 3′ end cleavage [13,22]. These isomiRs provide an ideal naturally occurring biological control, as the centered sites are shared between a miRNA and its isomiR, whilst the seed sites differ (Figure 1C). We show that imperfect-centered sites are common mediators of mRNA interactions, a finding that may explain the evolutionary conservation of the central region of miRNAs, and that will be important for decoding miRNA-regulated genetic networks.

## Results

### Biotin pull-downs identify hundreds of miRNA-mRNA interactions

To identify direct miRNA targets, we transfected 4 × 10^6^ HEK293T cells with 200 pmols synthetic biotinylated miRNA duplexes (Table S1 in Additional file 1). This very low ratio of duplex to cells was critical to avoid some of the problems with introducing an exogenous molecule on miRNA-mRNA stoichiometry, and we confirmed that transfection at this level made no substantial alterations to endogenous transcriptional networks (Figure S1A in Additional file 2). We then performed streptavidin purifications, and profiled the captured mRNA population via microarrays. At a 5% false discovery rate (FDR; see Materials and methods), between 963 (miR-10b-iso) and 2,261 (miR-182) microarray probes detected significantly higher expression in the biotin pull-down samples than in the mock-transfected controls (Table 1). The distribution of fold-changes observed for each miRNA duplex is plotted in Additional file 3. We considered the Ensembl (V62) transcripts with exact matches to these probes to be putative targets of the miRNA, identifying an average of 1,572 target genes per miRNA (Table 1). These numbers correspond well to those obtained by other experimental approaches [12,23,24].

**Table 1:** Number of inferred targets for each miRNA tested

**miRNA**	**Probes**	**Transcripts**	**Genes**
*miR-10a*	2,206	5,963	1,887
*miR-10a-iso*	1,648	4,211	1,468
*miR-10b*	1,588	3,940	1,365
*miR-10b-iso*	963	2,235	889
*miR-17-5p*	1,223	2,862	1,137
*miR-17-5p-iso*	1,656	3,731	1,461
*miR-182*	2,261	6,423	2,008
*miR-182-iso*	1,569	4,316	1,444
*miR-23b*	2,248	5,383	1,990
*miR-27a*	2,334	5,310	2,069

Probes: number of probes significantly enriched in pull-downs compared to controls (5% FDR). Transcripts: number of transcripts to which those probes map exactly. Genes: number of genes from which those transcripts originate.

### Predicted and validated targets are significantly enriched in the biotin pull-downs

Luciferase assays are usually considered to be the ‘gold standard’ for validating miRNA-mRNA interactions. We examined the concordance between our biotin pull-down results and miRNA targets that have been confirmed by luciferase assays in the literature (Table S2 in Additional file 1). We excluded genes that were not targeted by an Illumina microarray probe (and therefore could not be detected by our biotin pull-down) or that were not expressed in HEK293T cells, leaving 93 genes for this analysis. Forty-three previously validated targets were enriched by biotin pull-downs (Table S2 in Additional file 1) in HEK293T cells, which was significantly more than expected by chance (Table S3 in Additional file 1; Fisher’s exact test (FET), *P*-value <1.01 × 10^-12^). Interestingly, a lower proportion of targets validated in non-HEK cell lines were confirmed by our biotin pull-down (43.2%) than targets validated in HEK cell lines (51.4%), suggesting that cell type-specific factors can lower the concordance between orthogonal validation methods.

**Figure 2:**
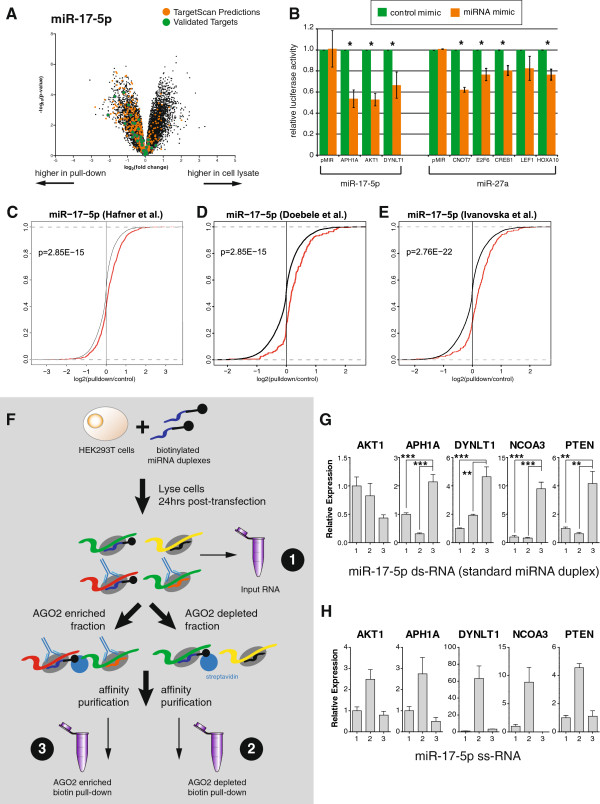
**Biotin pull-downs identify *bone fide *miRNA targets. (A)** Volcano plot showing the significance of the difference in expression between the miR-17-5p pull-down and the mock-transfected control, for all transcripts expressed in HEK293T cells. Both targets predicted by TargetScan or validated previously via luciferase assay were significantly enriched in the pull-down compared to the controls. **(B)** Results from luciferase assays on previously untested targets predicted using TargetScan and uncovered using the biotin pull-down. The plot indicates mean luciferase activity from either the empty plasmid or from pMIR containing a miRNA binding site in the 3′ UTR, relative to a negative control. Asterisks indicate a significant reduction in luciferase activity (one-sided *t*-test; *P* < 0.05) and error bars the standard error of the mean over three replicates. **(C-E)** Targets identified through PAR-CLIP or through miRNA over-expression studies show greater enrichment in the pull-down. Cumulative distribution of log fold-change in the pull-down for transcripts identified as targets by the indicated miRNA over-expression study or not. Red, canonical transcripts found to be miR-17-5p targets in the indicated study (Table S5 in Additional file 1); black, all other canonical transcripts; *p*, one-sided *P*-value from Kolmogorov-Smirnov test for a difference in distributions. **(F)** To confirm that our results were dependent on RISC association, cells were transfected with either single or double-stranded synthetic miRNAs, then subjected to AGO2 immunoprecipitation. The biotin pull-down was performed in the AGO2-enriched and AGO2-depleted fractions. **(G-H)** Quantitative RT-PCR revealed that, with double-stranded (ds) miRNA **(G)**, four out of five known targets were enriched relative to input mRNA (*P ≤ 0.05, **P < 0.01, ***P < 0.001) in the AGO2-enriched but not in the AGO2-depleted fractions, but this enrichment was not seen for the cells transfected with a single-stranded (ss) miRNA **(H)**. The numbers on the x-axis correspond to those in Figure 2**F**. Error bars represent the standard error of mean (sem).

As a second control, we examined the number of bioinformatically predicted targets in our biotin pull-downs. Although bioinformatic predictions have many false positives (and an as yet undefined level of false negatives) [6,8-10], we would still expect to see an enrichment for predicted targets in each of our biotin pull-downs. Importantly, predictions can be made for the isomiRs used in this study, for which very few luciferase assays (or other experimental validation of targets) have been performed. We used TargetScan [1] to predict targets for each miRNA and isomiR, and compared each list to the significantly enriched genes in the corresponding pull-down. In each case, we observed an enrichment of TargetScan-predicted genes in the pull-down fraction (Figure 2A; Figure S1B,C in Additional file 2), and the overlap was significantly more than expected by chance (Table S3 in Additional file 1; FET, *P* < 9 × 10^-180^). We selected eight previously untested targets of miR-17-5p (3) and miR-27a (5) for experimental validation using luciferase assays. TargetScan-predicted binding sites were cloned into the 3′ UTR of a luciferase reporter construct, and co-transfected into HEK293T cells with either their predicted miRNA mimic or a control mimic. We found that seven of eight target sites had significantly (*P* ≤ 0.05) reduced luciferase activity in the presence of a miRNA mimic compared to the control mimic (Figure 2B). As the rate of validation seen here (87.5%) greatly exceeds the validation rate of TargetScan predictions alone (39%) [8], this provides substantial experimental evidence that the biotin pull-downs are enriching for genuine and direct targets of the miRNAs.

As a third control, we compared the biotin pull-down results to those obtained using both PAR-CLIP [12] and miR-17-5p over-expression studies [23,24]. The PAR-CLIP experiment was conducted in HEK293 cells, which differs from the HEK293T cells used in our experiments by the exogenous expression of the SV40 large T antigen. This protein binds to and inactivates the p53 transcription factor [25], so the gene expression patterns between these cell lines differ. The over-expression experiments were also performed in very different cell lines (human umbilical vein endothelial cells (HUVECs) [23] and HCT116 cells [24]), and therefore we do not expect a perfect overlap between the targets detected between these orthogonal methods. By comparing the distribution of the miR-17-5p fold-change observed in this study between the transcripts that were detected as targets in previous studies and those that were not, we observed a significantly greater fold-change in the corresponding biotin pull-down in the former class (Figure 2C-E; Additional file 4; Kolmogorov-Smirnov tests, *P*-values from 1 × 10^-2^ to 1 × 10^-22^). Additionally, the overlaps between our inferred miRNA target sets and those deduced from the other studies were significantly greater than expected by chance (Table S3 in Additional file 1; FET, *P*-values from 4.68 × 10^-2^ to 5.17 × 10^-20^), although the enrichment was modest (odds ratio (OR) = 1.85; 95% confidence interval (CI) 1.7 to 2.1). Finally, although biotin-labeled miRNAs have been previously shown to incorporate into RISC [17], we wanted to be sure that our results were dependent on RISC association, and not due to cytoplasmic binding of free biotin-labeled miRNAs. To test this, we performed a hybrid AGO immune-precipitation and biotin pull-down (Figure 2F). HEK293T cells were transfected with either a standard biotinylated, double-stranded miR-17-5p miRNA duplex, or with a biotinylated single-stranded miR-17-5p sequence. Cells were lysed and subject to AGO immune-precipitation, creating an AGO2 enriched and AGO2-depleted fraction. Each fraction was then subject to a biotin pull-down, and the relative enrichment compared to input RNA (Figure 2F) was measured by quantitative RT-PCR (qRT-PCR). We specifically assessed the levels of the three novel seed sites identified above (Figure 2B) and two previously validated miRNA binding sites [8]. As endogenous RISC loading preferentially requires double-stranded miRNA duplexes [26,27], we would expect double-stranded but not single-stranded molecules to enrich for targets in an AGO2 precipitation. Four of five targets were found to be enriched in the AGO2 enriched fraction when a double-stranded molecule was used (Figure 2G), and but not when a single-stranded molecule was used (Figure 2H). This, when considered along with the data presented above, confirms that the biotin pull-down is consistently enriching for genuine targets of miRNAs, although we do not know if or how these interactions result in target repression.

### Biotin pull-downs are less likely to enrich for destabilized mRNAs, but have low rates of false positive target identification

If we are to use the biotin pull-downs to characterize non-seed site interactions, then it is important to understand the limitations of this protocol. To start characterizing the potential false negatives of the biotin pull-down approach, we selected six genes that we had previously reported to be targets of miR-17-5p that were not enriched in our pull-down [8]. We used qRT-PCR to determine whether these mRNA targets were destabilized by miR-17-5p and therefore less likely to appear as significantly enriched in the biotin pull-down. After transient transfection with biotinylated miR-17-5p (replicating the conditions of the biotin pull-down) we observed significant degradation of five of the six genes (Additional file 5). This result suggests that mRNA degradation probably affects whether or not a target can be detected via the biotin pull-down approach, and suggests that the concordance between biotin pull-down and luciferase assay could be higher when only comparing targets that are translationally regulated.

**Figure 3:**
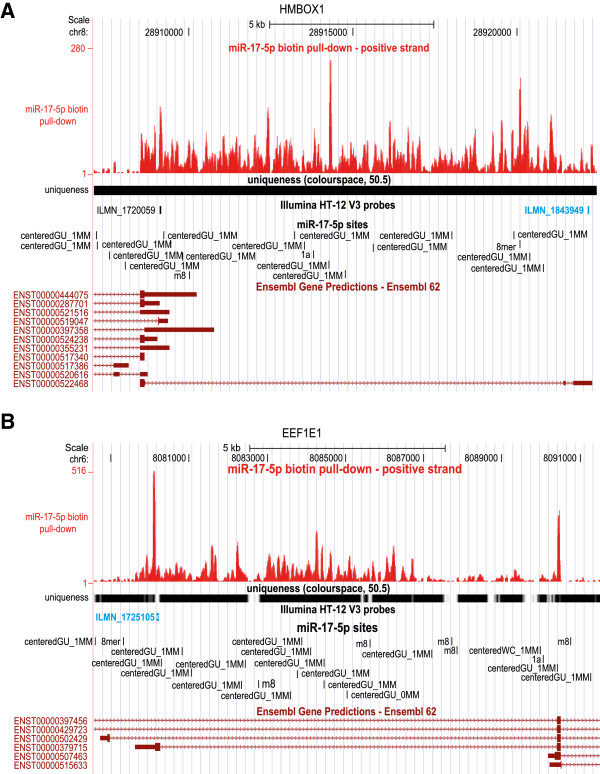
**miRNA binding in unannotated exons.** Sequencing the RNA captured in the miR-17-5p pull-down revealed several examples of targeting outside annotated exons. The red wiggle plot along the top indicates the number of reads from the RNAseq library that mapped at each position. Illumina microarray probes highlighted in blue showed significant enrichment in the biotin pull-down. **(A,B) ***HMBOX1 ***(A)** encodes a homeobox-containing transcription factor and *EEF1E1 ***(B)** a translation elongation factor. Probes detecting these genes were significantly enriched in the pull-down (adjusted *P*-values: ILMN_1843949, 0.002; ILMN_1725105, 0.03). None of the annotated exons (maroon boxes) contained predicted miR-17-5p sites. However, the RNAseq data suggested that both genes had retained introns that resulted in a longer 3′ UTR, and this region did contain binding sites.

We then wished to examine the impact of transcriptional complexity on our interpretation of the microarray results. Specifically, we were curious about the subset of transcripts (average 19%) that were significantly enriched in the pull-down but that had no predicted binding sites (either seed sites or any type of centered sites, discussed below) in their annotated exons. We hypothesized that this set may contain examples of unannotated exons and that this could lead to the incorrect classification of these targets as false positives. To investigate this possibility, we performed strand-specific RNAseq from the RNAs captured in the biotinylated miR-17-5p pull-down experiment. We examined all 26 HUGO genes that were targeted by an Illumina probe that was significantly enriched in the pull-down, that had exact matches only to transcripts with no predicted miR-17-5p seed and perfect or imperfect centered binding sites, and that predominantly spanned unique genomic regions to which the sequencing reads could be mapped [28]. Of these, eight clearly had either extended 3′ UTRs (*HMBOX1*, *TBPL1*, *ZNF786*, *NDUFAF3*) or retained introns (C2orf34, *COQ10B*) or both (C16orf68, *EEF1E1*), and, in all cases, these extra regions contained miR-17-5p seed or centered sites (Figure 3). Two additional genes (*ERLEC1*, *UBB*) showed weak evidence for retained introns or extended 3′ UTRs that contained miR-17-5p sites. This suggests that almost 40% of the genes that we had initially assumed to be false positives were in fact likely to be true targets.

### Hundreds of miRNA-mRNA interactions are mediated by centered sites

Having confirmed to our satisfaction that the biotin pull-downs could provide a high level of sensitivity for examining non-seed mediated interactions, we then searched for putative seed and perfect or imperfect centered binding sites (Figure 1C; Additional file 6) of each of our 10 miRNAs in the transcripts detected by the microarray probes. We asked what proportion of putative miRNA targets could be explained by the presence of these sites (Additional file 7). Studies that measured the change in protein output after miRNA transfection found that 50 to 70% of down-regulated proteins (log2 fold-change < -0.5) had a seed site [6,10] for the relevant miRNA. Hafner *et al.*[12] reported that 50% of their PAR-CLIP clusters contained a seed site for one of the top 100 most expressed miRNAs in HEK293 cells. We found that, on average, 41.3% (standard deviation 10.1%) of significantly enriched transcripts contained one or more seed sites for the biotinylated miRNA. When centered sites were considered, allowing for GU wobble and up to one mismatch, we could account for substantially more of the significantly enriched targets (mean 82.7%, standard deviation 7.5%), suggesting that centered sites could indeed bind mRNAs on a large scale.

Shin *et al.*[21] reported that centered site matches were significantly associated with repression only if they involved perfect Watson-Crick base-pairing. However, we postulated that these sites might still be able to mediate interactions if they included GU pairs or a mismatch. Thus, in our analysis, we considered centered sites with only Watson-Crick binding (centered-WC) or with GU wobble (centered-GU) and with either no mismatches (0MM) or with one mismatch (1MM) (that is, any base pair other than C-G, A-U or G-U). Centered sites with only Watson-Crick matches accounted for only a small proportion of the significantly enriched transcripts (0.5% and 6.6% for 0MM and 1MM sites, respectively), but a much greater proportion of these putative target transcripts contained centered-GU sites (12.5% and 72%); thus, these were far more prevalent than seeds in our data set.

**Figure 4:**
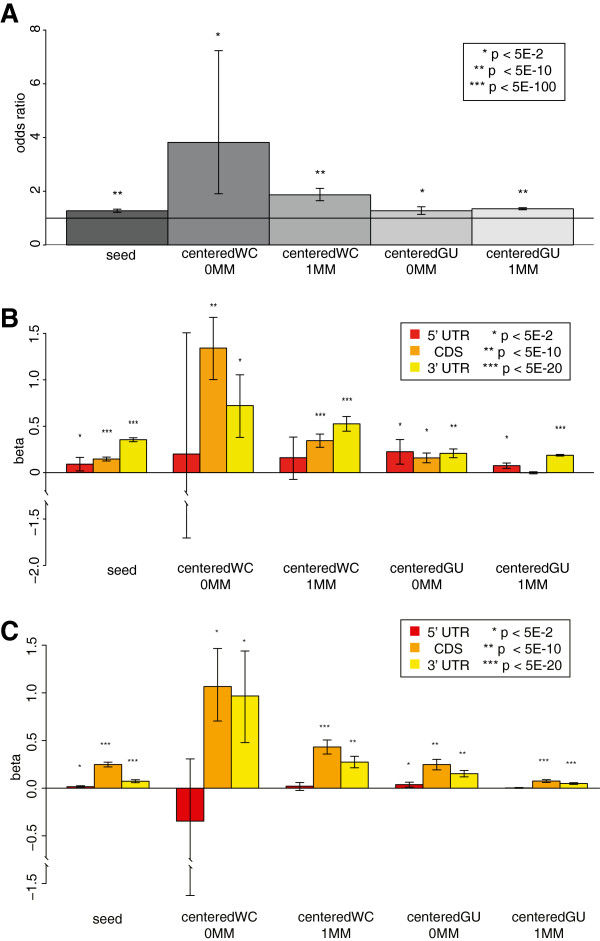
**Transcripts enriched in the pull-down were enriched for centered sites as well as seeds. (A)** The odds ratios from Fisher’s exact tests for an enrichment of transcripts with the indicated site type amongst the putative target set. Transcripts with multiple different types of site were excluded. **(B,C)** The coefficient estimates from logistic regressions of enrichment status on site count. Error bars indicate 95% confidence intervals. Tests were based on data from all 10 pull-downs combined. In **(C)**, transcripts with multiple different types of site were excluded. CDS, coding sequence.

As expected, transcripts with seed sites were significantly over-represented in the putative target set compared to the non-target set when considering transcripts with only a single type of site (FETs; Figure 4A). Interestingly, the same significant over-representation was also seen for centered sites (with or without GU wobble or one mismatch), suggesting that they, too, might be mediating miRNA-mRNA interactions. To ensure that this was reproducible, and not an artifact of small sample size, we repeated our analysis using all transcripts enriched in our pull-downs. For every site type, we again saw a significant over-representation of transcripts with a site amongst the putative target set, with the strongest enrichment being seen for the centered sites with perfect Watson-Crick pairing (Figure S7A in Additional file 8). These results suggest that the centered sites of the miRNA can mediate interactions with transcripts even in the absence of seed sites.

### Centered sites can mediate interactions in the coding sequence and 3′UTR of mRNAs

To investigate whether the location of the putative miRNA binding sites affected whether or not a transcript would be enriched in the pull-down, we fitted a logistic regression model for enrichment status, including the counts of each type of site within different transcript regions as covariates (see Materials and methods). This model was highly significant (*P* < 0.0001), although it only explains a small fraction of the variance (Nagelkerke r^2^ = 0.04). As expected, the more seed sites there were in a transcript’s 3′ UTR, the more likely it was to be significantly enriched in the pull-down (Figure 4B, left-most yellow bar). Interestingly, all types of centered sites in the 3′ UTR were significantly associated with enrichment status (Figure 4B, yellow bars). Consistent with recent literature [6,20,29-32], most site types were significant in the coding sequence (CDS) too (Figure 4B, orange bars), although the effects were strongest in the 3′ UTR in most cases. Curiously, when we controlled for the length of the regions by fitting the logistic regression with site densities as covariates, the coefficient estimates were greater for the CDS than the 3′ UTR (Figure 4C) for all site types. It may be that while miRNAs are more likely to target UTRs with more sites, there is a trade-off between the number and accessibility of sites (reflected by site density), and site accessibility is more important in the UTR than the CDS, perhaps because of mRNA secondary structure. Interestingly, though, the r^2^ was much lower for this model (0.008) than when we fit site counts as covariates, although it was still significant (*P* < 0.0001). To ensure that the logistic regression was not being confounded by the inclusion of multiple transcripts bound by the same probe (which are not independent of one another), we ran the analysis again including only the canonical isoform of each gene. This did not substantially change the results (*P* < 0.0001, Nagelkerke r^2^ = 0.05 for site count; *P* < 0.0001, Nagelkerke r^2^ = 0.009 for site density; Figures S7B,C in Additional file 8). We conclude that centered sites can mediate interactions both within and outside the 3′ UTR, although 3′ UTR interactions are more important, as has previously been shown for seed sites [33-35].

### Similarity between target sets of canonical miRNAs and their isomiRs

If centered sites are indeed mediating biologically relevant interactions, then we would expect this to be reflected in the pattern of transcripts that are enriched in the biotin pull-down of both a canonical miRNA and its isomiR.

**Figure 5:**
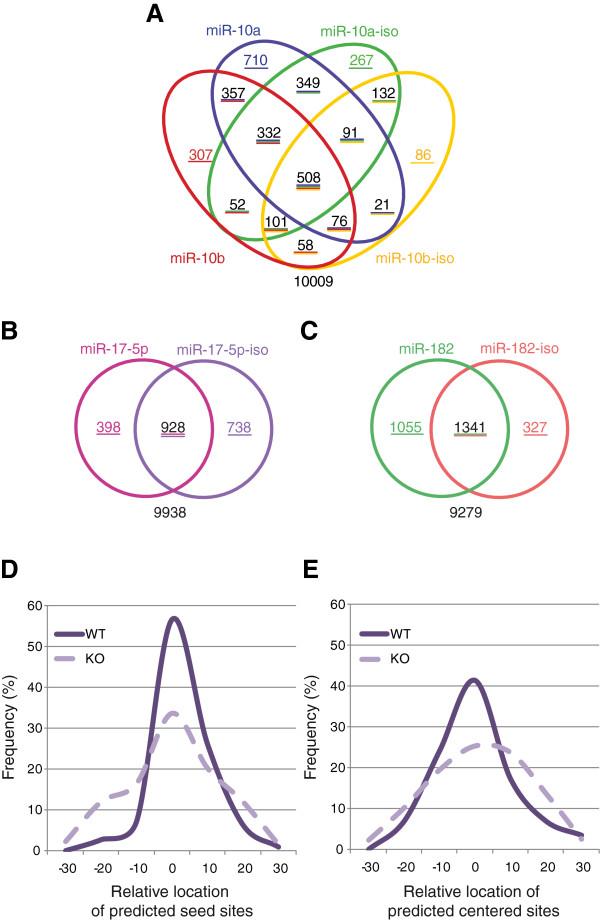
**IsomiRs and canonical miRNAs target many of the same transcripts. (A-C)** Venn diagrams indicating the number of microarray probes significantly enriched in each biotin pull-down and overlapping between related miRNAs. **(D,E)** Distribution of the location of predicted seed sites **(D)** and centered sites **(E)** relative to the center nucleotide of AGO2 peaks in mice wild-type for miR-155 (WT, solid line), or in miR-155 knock-out mice (KO, dashed line). AGO2 peaks were previously identified by Loeb *et al.*[20].

Since the isomiRs have different seed sites from their canonical partners but share overlapping centered sites, we might expect the transcripts they target in common (Figure 5) to be enriched for centered sites relative to the transcripts targeted by only one or the other. Conversely, since miR-10a and miR-10b have the same seed but different centered sites, the transcripts targeted by both miRNAs might be enriched for seeds compared to transcripts targeted by only miR-10a or miR-10b. For this analysis, we excluded transcripts with multiple different types of sites for the same miRNA. Although this approach lost some statistical power through the large reduction of transcripts, we still found statistical support (*P* ≤ 0.05) for each of the five comparisons (miR-10a versus miR-10b; miR-10a versus miR-10a-iso; miR-10b versus miR-10b-iso; miR-182 versus miR-182-iso; miR-17-5p versus miR-17-5p-iso), and a total of 14/20 of the observed enrichments confirmed our hypothesis (Table S4 in Additional file 1). For example, the transcripts that were enriched in the pull-downs of both miR-182 and miR-182-iso were significantly more likely to have a centered site compared to the set enriched in only the miR-182 pull-down (one-sided FET; OR = 1.45; 95% CI 1.04 to 2.04; *P* = 1.9 × 10^-2^) or in only the miR-182-iso pull-down (OR = 2.07; 95% CI 1.54 to 2.8; *P* = 9.4 × 10^-7^). There was also a significant over-representation of transcripts with a seed site amongst the transcripts targeted by miR-10a and miR-10b compared to those targeted by only miR-10a (OR = 2.13; 95% CI 1.3 to 3.6; *P* = 2.0 × 10^-3^). These results again support the hypothesis that centered sites in mRNAs can mediate interactions with miRNAs, although we cannot rule out the possibility that the transcripts pulled down by multiple miRNAs were being targeted by non-canonical sites other than the centered site.

### Centered sites are enriched in AGO-bound regions

If centered sites are truly mediating miRNA-mRNA interactions, and are not an artifact of the experimental approach used here, then we would expect such sites to be enriched in regions of mRNAs bound by RISC and endogenous miRNAs. In 2012, Loeb *et al.*[20] used differential AGO2 HITS-CLIP to profile murine primary T cells that were either miR-155 wild-type or were miR-155 deficient due to genetic knock out. Due to the high number of biological replicates (12 of each condition), the authors were able to robustly define 14,634 AGO2 bound regions, with 398 of these differentially detected only in wild type compared to knock out (adjusted *P* < 0.05), and therefore likely to be directly mediated by miR-155. We exploited these data to look for the frequency and location of imperfect centered sites and seed sites relative to the peak. We first confirmed that seed sites were significantly enriched in differentially detected AGO peaks compared to AGO peaks that were unchanged between wild type and knock out (FET, *P* ≈ 8.07 × 10^-32^, n = 111), and were significantly skewed towards the center of the predicted peak (excess kurtosis = 7.34; Figure 5D). We also observed enrichment and kurtosis for imperfect centered sites (FET, *P* ≈ 5.19 × 10^-3^, n = 29; excess kurtosis = 3.50; Figure 5E), although these sites were less frequent than seed sites. The overlap between seed and centered sites (n = 6) was not significantly different to what would be expected by chance (FET, *P* = 0.7887). Together these results suggest that imperfect centered sites can mediate endogenous miRNA-mRNA interactions independently of seed sites.

### Imperfect centered sites primarily suppress target mRNAs through translational inhibition

**Figure 6:**
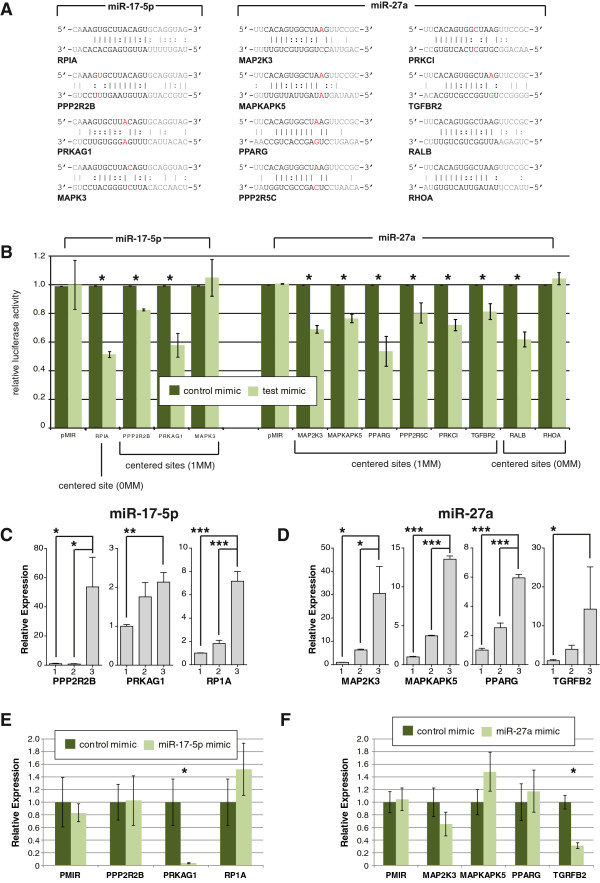
**Centered sites can repress gene expression. (A)** Four imperfect centered binding sites for miR-17-5p (left column) and eight for miR-27a (right columns) were tested via luciferase assay. Vertical line, perfect Watson-Crick matches; colon, GU wobble sites; shaded bases, regions outside of the central zone (nucleotides 3 to 15) that may participate in binding; red text, mismatches between the miRNA and its target in the central zone. Note that no other binding sites of any type were predicted within these mRNAs. **(B)** Mean luciferase activity relative to negative controls from the empty plasmid (pMIR) or pMIR containing one of the imperfect binding sites described above in the 3′ UTR. Asterisks indicate significant reduction in luciferase activity (one-sided *t*-test; *P* < 0.05), error bars represent standard error of the mean over three replicates. **(C,D)** A hybrid AGO2 immunoprecipitation/biotin pull-down was performed as in Figure 2F, and qRT-PCR was used to detect mRNAs confirmed to be targets of miR-17-5p **(C)** and miR-27a **(D)** above. Numbering on the x-axis refers to the samples described in Figure 2F. All targets were found to be significantly enriched in the AGO2-enriched fraction (*P ≤ 0.05, **P < 0.01, ***P < 0.001). **(E,F)** Analysis of luciferase expression levels by qRT-PCR found that only one out of three imperfect centered sites for miR17-5p **(E)** and one out of four sites for miR-27a **(F)** showed significantly reduced luciferase mRNA levels (*P* < 0.05, indicated by asterisks), indicating that most interactions that result in reduced protein are due to translational inhibition and not mRNA degradation.

Although our results suggest that imperfect centered sites can direct RISC to mRNAs, it does not necessarily follow that those sites can mediate repression of gene expression. To address this specific question, we randomly selected 12 genes that were both significantly enriched in the biotin pull-down experiment and had only a single centered miRNA binding site in the transcript, for testing via luciferase assay (Figure 6). All but one of the centered sites included GU wobble, and 9 of them also had a single mismatch between the miRNA and the mRNA. We validated two of three centered sites with no mismatch, and eight of nine centered sites with one mismatch. The rate of validation seen here (79%) is much higher than previously reported validation rates based on bioinformatics predictions alone (39%) [8], and again supports the robustness and utility of the biotin pull-down approach. These results demonstrate that even without perfect complementarity, centered sites can mediate mRNA repression in mammalian systems.

To ensure that the centered sites were mediating their function through RISC, we repeated the hybrid AGO immune-precipitation/biotin pull-down approach (Figure 2F) to look for enrichment of these centered sites in AGO2-RISC. For both miR-17-5p imperfect centered sites (Figure 6C), and miR-27a imperfect centered sites (Figure 6D), all targets were found to be significantly enriched (*P* ≤ 0.05) in the AGO2 enriched fraction compared to the input mRNA population, demonstrating that interactions between an imperfect centered site and its target mRNA were mediated through RISC.

Finally, to determine whether imperfect centered sites mediate mRNA destabilization or translational inhibition, we repeated the luciferase assays described above (Figure 6B), and measured the level of luciferase gene mRNA via qRT-PCR. Changes in the mRNA levels between the control mimic and the test miRNA would indicate mRNA destabilization, whereas no changes in the mRNA levels would indicate translational inhibition. Only one out of four sites for miR17-5p (Figure 6E) and one out of five sites for miR-27a (Figure 6F) showed significant (*P* < 0.05) reduction of mRNA levels, indicating that most interactions that result in reduced protein are due to translational inhibition rather than mRNA degradation, although both types of regulation are possible. These experiments together provide substantial experimental evidence that imperfect centered sites are common targets of miRNAs, and mediate functional interactions.

### The biotin pull-down technique is slightly biased against seed sites

A consideration for interpreting the results derived from this protocol is that the biotin tag on the synthetic miRNA duplexes may prevent their 3′ ends from binding to the PAZ domain of Ago [2]. This may allow biotin-labeled miRNAs to interact more easily with target mRNAs and alleviate the requirement for 5′ end binding. The method may therefore be biased towards detecting interactions that are not mediated by the canonical seed.

**Figure 7:**
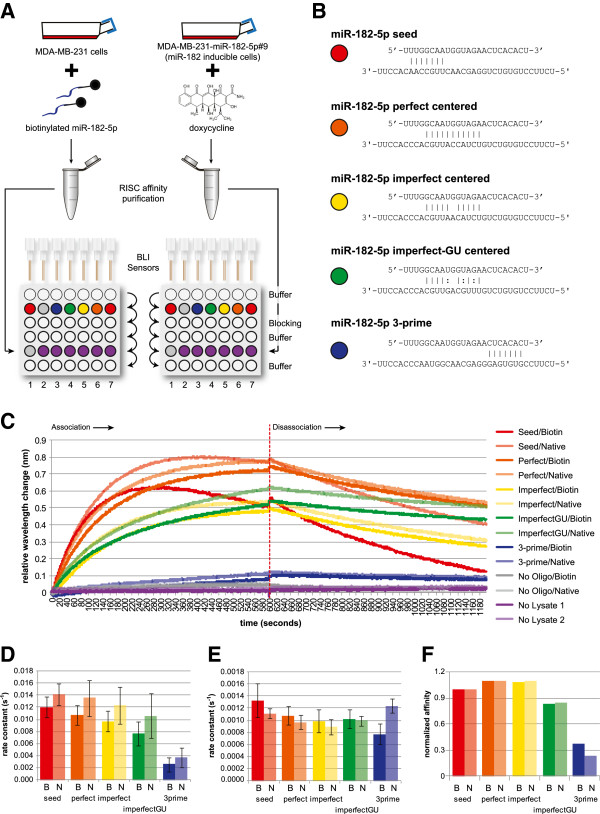
**Biotin RISC differs only slightly from native RISC in affinity for centered sites. (A)** Schematic diagram showing the bio-layer interferometry (BLI) experimental design. Cells were either transfected with biotinylated miR-182-5p or induced to over-express miR-182-5p, and RISC complexes containing these miRNAs were affinity purified. The two RISC solutions were used in parallel BLI runs to detect association and disassociation with RNA oligos containing the binding sites. The arrows indicate the sequential immersion of the sensors in a row. Lane 1, no lysate; lane 2, no oligo; lane 3, miR-182-5p 3 prime binding site; lane 4, miR-182-5p imperfect centered site with GU wobble; lane 5, miR-182-5p imperfect centered site; lane 6, miR-182-5p perfect centered site; lane 7, miR-182-5p seed site. **(B)** The binding between miR-182-5p and the RNA oligo is illustrated. The miRNA is depicted on the top strand, and the binding site in the oligo on the bottom strand. Perfect Watson-Crick matches are indicated with the vertical line, and GU wobble sites by a colon. **(C)** A representative association/disassociation curve from the BLI showing the wavelength interference (nm) versus time. The dashed line indicates the time at which the BLI sensors were removed from the RISC solution and transferred to buffer. **(D)** Rate constants for association were calculated independently from four biological replicates, and plotted separated for biotin RISC **(B)** and native RISC (N) across all five binding sites. Error bars represent standard error of the mean (sem). **(E)** Rate constants for disassociation were calculated and plotted as for **(D)**. Error bars represent standard error of the mean (sem). **(F)** The normalized affinity for each site type was calculated by dividing the association rate constant by the disassociation rate constant, and normalizing all site types to the ratio for the seed site. This shows that, with the exception of 3' sites, the relative affinities between biotin RISC and native RISC are essentially equivalent.

To explore the potential bias of this method in the affinity for different types of target sites, we used bio-layer interferometry (BLI) to detect the rates of association and disassociation of RISC with various types of binding sites (Figure 7A). The higher the ratio of association/disassociation is, the stronger the affinity of RISC to a binding site will be. Biotin miR-182-5p RISC was generated by transfecting MDA-MD-231 cells with biotin-labeled miR-182-5p duplexes, whilst native miR-182-5p RISC was generated by inducing expression of miR-182-5p by adding doxycycline to a previously generated and validated inducible cell line [36]. RNA oligos containing seed, centered, and 3′ binding sites (Figure 7B) were labeled with biotin and bound to the streptavidin-coated BLI sensors, blocked in free biotin blocking buffer, and used to probe biotin RISC or native RISC. The 'no oligo' control confirmed the effectiveness of this blocking and confirmed that there was no association between the biotin RISC and the BLI sensors after blocking (Figure 7C). The rate constants for association and disassociation were calculated independently for four independent biological replicates, and compared between biotin RISC and native RISC (Figure 7D,E).

As expected, the association rates for seed sites are the highest of all sites, and dramatically higher than for 3' sites. Although we find no statistically significant difference between native RISC and biotin RISC for any interaction, we did observe a trend for native RISC to associate with all binding sites faster than biotin RISC (Figure 7D) and for native RISC to disassociate slower (Figure 7E) than biotin RISC. The only exceptions were imperfect-GU sites, which showed the same dissociation rate for native and biotin RISC, and 3' sites, which were much higher for native risk (approaching statistical significance, *P* ≈ 0.06). Thus, all but 3' site-mediated interactions are stronger in the absence of the biotin tag. Overall affinities (as measured by the ratio of association/disassociation) are very similar between biotin and native RISC for all types of sites (Figure 7F), but it is worth noting that the protocol used here could be affected by the higher disassociation rates of the seed sites. This is because in the biotin pull-down, unlike CLIP-based methods, the interactions are not fixed in the cell before isolation, so they can disassociate, and we would expect biotin RISC to disassociate more quickly from seed sites than native RISC. Together, this could result in a reduced affinity, transcriptome-wide, for seed sites in our biotin pull-down compared to what would be expected for native RISC-based protocols. We see some evidence for this in the slightly lower proportion of targets that are accounted for only by seed sites in our study compared to other studies - approximately 41% of our biotin pull-down targets can be explained by a seed site, versus 50% for PAR-CLIP data [12] (although we note that this 50% refers to a seed match to any of the top 100 expressed miRNAs). We interpret these results to mean that the biotin pull-down has a slight bias away from seed sites rather than any particular bias towards centered sites. In our data, this probably manifests as a lower percentage of seed sites, but importantly, the affinity between native RISC or biotin RISC to centered sites is essentially equivalent and therefore our conclusions regarding the biological relevance of these sites are unlikely to be an artifact of the protocol used, although the prevalence of these interactions may be slightly inflated by the bias away from seed sites.

## Discussion

In order to understand the function of a particular miRNA, it is essential to know its targets. All methods for elucidating these have weaknesses, including the biotin pull-down approach described previously [17,18] and used here. We chose this protocol because it is not confounded by secondary effects (unlike microarray-based miRNA modulation studies) and because it is more sensitive for detecting differences between two closely related miRNAs than other whole-transcriptome approaches such as HITS-CLIP. The inclusion of stringent bead-blocking and washing steps has allowed us to substantially increase the specificity and dynamic range of the assay; the protocol described by Orom *et al*. [18] achieved a maximum fold-change of 4, while the transcripts in our biotin pull-downs were enriched up to eight-fold (Figure S1B in Additional file 2). However, we should anticipate that a proportion of transcripts detected as significantly enriched in our biotin pull-down may still not interact with the miRNA of interest. This type of false positive could occur through non-specific interaction with the magnetic streptavidin beads, or by association with endogenous biotin rather than with a biotinylated miRNA. Several lines of evidence suggest that this type of false positive is reasonably uncommon: (i) up to 90% of significantly enriched transcripts contain a predicted binding site (average 82.7%); (ii) of the remaining annotated (HUGO) transcripts without predicted binding sites, almost 40% were shown to contain novel transcriptional events with predicted bindings sites within them; and (iii) the vast majority of miRNA binding sites tested in luciferase assays were validated (Figures 2B and 6B) and were enriched in AGO2 containing silencing complexes (Figures 2G,H and 6C,D), although this was a small number of sites.

A second class of false positives would be those interactions that genuinely involve the biotinylated miRNAs but that do not occur endogenously in cells. This set is more difficult to define in the absence of a large cohort of true positives - even the ‘gold standard’ luciferase assays involve exogenous plasmids tested with exogenous miRNA mimics or inhibitors. While these assays can show that it is possible for a specific miRNA to interact with a specific binding site, they cannot prove that a specific miRNA interacts with a specific binding site endogenously. Factors such as RNA secondary structure, differences in the cellular concentration of miRNA and other miRNA binding factors, and secondary effects of miRNA modulation can all confound the interpretation of what are 'true targets' of a given miRNA. Just as precise false positive rates are difficult to calculate, the false-negative rates are equally intractable. An accurate assessment of the false negative rate would require a very large number of luciferase assays (if these are considered the gold standard) to establish a true positive set, and then ask how many of these were missed by the biotin pull-down approach. Such an experiment is impractical and expensive. However, like any difficult biological problem, orthogonal validation and careful interpretation are critical, and we were encouraged that miRNA targets inferred from all high-throughput methods and luciferase assays were significantly more likely to be enriched in our pull-downs than other genes (Table S2 in Additional file 1), and that the overlap seen between our results and those from the miR-17-5p over-expression experiments is similar to that observed between the latter and AGO2 immuno-precipitation datasets [34,37].

Although we did not find any statistically significant differences between native RISC and biotin RISC in their affinities to seed or centered sites, the trend did suggest that biotin-labeled miRNAs were slightly less able to detect seed sites than native miRNAs, which could result in a relative increase in the proportion of centered sites in our data. This could explain the drop from 50% seed sites in the PAR-CLIP data [12] to 41% in our study. However, the large variability amongst individual miRNAs is likely to be a substantial contributing factor - we observed a standard deviation of 10.1% in the proportion of transcripts containing seed sites. Similarly, although imperfect centered sites were significantly enriched in endogenous AGO2 clusters mediated by miR-155 [20], they were almost four times less frequent than seed sites (7.3% versus 27.9%, respectively). This difference is likely to be a combination of the bias of the biotin pull-down approach, unexplored biases in the HITS-CLIP detection (including cluster stringency and bioinformatic inference of sites), and genuine biological differences in the targeting profiles of different RISC species. Regardless, while any bias towards site types should be considered when interpreting their relative importance, it is clear that imperfect centered sites account for a substantial fraction of the total interactions between the miRNA and mRNA populations.

We have used the biotin pull-down technique to investigate associations between the number of predicted miRNA-binding sites and a transcript’s enrichment in the pull-down (Figure 4). These associations were stronger for site counts in the 3′ UTR than in the CDS, consistent with previous reports that 3′ UTR seed matches are more important for mediating interactions [33-35]. It is striking that the number and density of centered sites, and not just of seed sites, was also significantly associated with enrichment in the pull-down. The hypothesis that centered sites mediate many interactions is also supported by the considerable differences between the target sets of miR-10a and miR-10b (Figure 5A), which share the same seed but have different centered sites, although it is possible that some differences are accounted for by 3′ complementary sites.

Shin *et al*. [21] reported that 'perfect 11-mer matches starting at miRNA positions 3, 4 and 5 were each significantly associated with repression' in over-expression studies, but that 11-mer matches with single mismatches or GU wobbles were not. However, microarray based-assays that rely on changes in the expression levels of target mRNAs will not identify translationally repressed mRNAs, which have been shown to be the primary direct effect of miRNAs [38]. Results from our luciferase assays, which detect miRNA effects at both the mRNA and protein levels, suggest that imperfect centered sites are *bona fide* miRNA-binding sites that can elicit repression. Given that only a small proportion of the imperfect centered sites result in mRNA destabilization (Figure 6E,F), this would explain why these sites failed to validate in microarray-based studies, but did validate here in luciferase assays (Figure 6B).

Further experimental work on a larger number of sites would be required to elucidate precisely the minimal length and maximum number of mismatches required for centered site-mediated interactions. Regardless, the finding that both perfect and imperfect centered sites can repress gene expression has significant implications for understanding miRNA-controlled genetic networks, as the prevalence of centered sites in the mammalian transcriptome dramatically increases the connectivity and complexity of the networks. It may also explain the evolutionary conservation of regions outside of the seed site [7,21], although we cannot formally exclude the hypothesis that these regions are conserved due to processes related to miRNA biogenesis. Further experimental work would also be required to compare the efficacy of seed versus centered sites. Although the percentage reduction in luciferase activity shown here (Figure 6B) is comparable with that seen in other studies for seed sites [8], a thorough comparison would require thousands of luciferase assays with accompanying RNAseq and proteomics data.

While centered sites will clearly need to be factored into systems biology models of genetic regulation, it is currently unclear how much (if any) predictive power would be added to target prediction algorithms by the inclusion of imperfect centered sites. It was beyond the scope of this study to develop a prediction model incorporating centered site information, although it seems likely that the high frequency of these sites in the transcriptome would require that additional information such as evolutionary conservation and site accessibility/RNA secondary structure be incorporated to reduce the rate of false positive predictions, as is the case for seed sites. Despite the conceptual simplicity of looking for regions of complementarity, miRNA target prediction suffers from both a lack of specificity (due to the short binding sites), and a lack of sensitivity due to the exclusion of known binding modalities to increase prediction accuracy. This is analogous to the problems suffered by transcription factor binding prediction software, and was a significant driver in the quest for unbiased experimental methods such as ChIP-seq. We believe that the biotin pull-down methodology is complementary to other transcriptome-wide tools such as HITS-CLIP to examine targets of miRNAs without prior expectation of binding modes.

While we have demonstrated here that centered sites are direct targets of miRNAs that result in molecular repression of the target, the larger relationship between targeting and functional repression remains an open question. In biological networks, mRNAs can be insulated from the effects of miRNAs by simply expressing more copies of the mRNA [8], so whilst individual transcripts are still targeted and repressed, the overall protein levels may not change. How many of the targets identified here are functionally repressed versus molecularly repressed is currently unknown, but this is not a weakness of the biotin pull-down (or indeed, a weakness of any other transcriptome-wide method). Instead, this presents us with the interaction data necessary to model properly the outcomes of biological networks in different cell types or tissues with different mRNA expression levels. It will be the combination of these transcriptomic methods with proteomics data that will enable the next leap in understanding.

## Conclusions

We used a biotin pull-down method to determine the direct targets of 10 human miRNAs, and found that imperfect centered sites are common mediators of miRNA-mRNA interactions. These sites can repress mRNA activity, typically through translational repression, but further work is needed to establish their functional importance relative to seed sites. Our findings may explain the evolutionary conservation of the central region of miRNAs, and they have important implications for studying miRNA-regulated genetic networks, and for interpreting putative disease-causing variants that do not alter protein sequence but that may fall inside miRNA binding sites.

## Materials and methods

### Biotin pull-downs, microarray hybridizations and sequencing

We optimized the biotin pull-down method of Orom *et al*. [18] to improve RNA yield and dynamic range. Briefly, 200 pmoles of synthetic biotin-labeled miRNA duplexes (Table S1 in Additional file 1) were transfected into 4 × 10^6^ HEK293T cells using HiPerFect Transfection Reagent (QIAGEN, Melbourne, VIC, Australia). Cells were harvested after 24 hours, and lysed in hypotonic lysis buffer (10 mM KCl, 1.5 mM MgCl_2_,10 mM Tris-Cl pH 7.5, 5 mM DTT, 0.5% NP-40, 60U/ML SUPERase•In (Ambion, Austin, TX, USA) and 1X Complete Mini protease inhibitor (Roche, Brisbane, QLD, Australia)). Cell debris was cleared by centrifugation (≥10,000 g at 4°C for 2 minutes). The supernatant was transferred to a clean tube, and NaCl was added to a final concentration of 1 M. myOne C1 Dynabeads (25 μl; Invitrogen) were pre-blocked with 1 μg/μl bovine serum albumin and 1 μg/μl yeast tRNA (Invitrogen), and incubated with the supernatant for 30 minutes at room temperature. Beads were then washed with hypotonic lysis buffer and 1 M NaCl before RNA extraction using an RNeasy Kit (QIAGEN) according to the manufacturer’s instructions. Pull-downs were conducted in duplicate or triplicate for each miRNA, and 50 ng of the captured mRNAs per sample were amplified and labeled using the Illumina TotalPrep RNA Amplification Kit (Invitrogen) as per the manufacturer’s instructions. Samples were profiled on Illumina Human HT-12 chips along with control RNA from mock-transfected cells to which no miRNA had been introduced. In parallel, RNA extracted from the miR-17-5p pull-down was used in RNAseq library construction as previously described [39] and 50 bp tags were generated on the Applied Biosystems SOLiD (Melbourne, VIC, Australia). These were mapped to the human genome (GRCh37) using the default parameters of the X-MATE pipeline [40]; 70,714,217 of the 101,137,997 tags aligned to GRCh37 (70%).

### Hybrid AGO immuno-precipitations and biotin pull-downs

We transfected cells with either synthetic biotinylated miRNA duplexes or synthetic single-stranded biotinylated miRNAs and lysed the cells as described above. After clearing the cell lysate, we harvested 10 μl to use as the input RNA sample. We then used a Dynabeads Protein G Immunoprecipitation Kit (Invitrogen) combined with a ChIP grade mouse monoclonal antibody to AGO2 (ab57113, Abcam, Cambridge, MA, USA) to perform the AGO enrichments according to the manufacturers’ instructions. Briefly, 50 μl of beads per precipitation were washed prior to incubation with 10 μg of antibody at room temperature for 10 minutes. Cell lysate was added to the bead-antibody complexes, and incubated for 10 minutes at room temperature. The supernatant from this step was saved as the AGO2-depleted fraction, and the beads were kept as the AGO2-enriched fraction. The beads were washed three times in 200 μl of wash buffer, prior to elution in 30 μl of elution buffer. We then completed the biotin pull-down protocol on both the AGO2-enriched fractions and the AGO2-depleted fractions, as described above.

### Primary analysis of microarray data

Statistical tests were conducted in R. Microarray data were normalized using the *lumi* package [41], by applying background adjustment, variance-stabilizing transformation [42] and robust spline normalization [43] successively. Hierarchical clustering of normalized data (Additional file 9) showed that replicates tended to cluster together and that the samples from the pull-down of a canonical miRNA clustered close to those from the corresponding isomiR pull-down, as expected given the similarity in their sequences. One miR-10b pull-down sample was excluded because it did not cluster with the other replicates. Microarray probes that detected expression above background (detection *P*-value <0.01) in all control samples were retained (n = 12,008). The lmFit and eBayes functions in the *limma* package [44] were used to test for differences in RNA abundance ('differential expression') between the pull-down samples and the controls. The FDR was calculated to account for multiple testing [45]. Probes that met the 5% FDR threshold (for one-sided tests) were considered significantly enriched in the pull-down. The transcripts (EnsEMBL V62) to which they matched exactly were considered putative targets of that miRNA.

### Analysis of published data on miRNA-mRNA interactions

These lists of targets were compared to results from published datasets, considering miRNA-target interactions that had been demonstrated by luciferase assays (Table S3 in Additional file 1) and by various high-throughput methods. Table S5 in Additional file 1 summarizes our analysis of the latter. To test whether the biotin pull-down targets were enriched in the target list from another study, a FET was applied to ask whether the biotin pull-down targets were more likely than non-targets to be in the target list from another study. Unless otherwise stated, reported one-sided *P*-values are from an upper-tailed test, and an OR >1 indicates that genes/transcripts/probes that are targets in our biotin pull-down are more enriched amongst the targets identified in the experiment being examined than those that are not targets in the pull-down.

Unless otherwise stated, reported one-sided *P*-values are from an upper-tailed test, which evaluates the hypothesis that the odds ratio, $\frac{w/x}{y/z}$, is greater than 1. Ambiguous genes/transcripts (those that were detected both by a probe that was significantly enriched and by a probe that was not) were excluded unless otherwise specified. Kolmogorov-Smirnov tests were conducted to compare the distribution of fold-change (expression in biotin pull-down/control) between canonical transcript targets inferred from published experiments and all other canonical transcripts (the canonical EnsEMBL transcript is the longest produced from a gene).

### *In silico* prediction of miRNA-binding sites

TargetScanS [1] was run on all EnsEMBL transcripts to find seed matches to the miRNAs of interest. A Perl script was also written to count matches to the centered site (11 bp sequence starting at position 3, 4 or 5 of the miRNA). For centered sites, we allowed GU wobble and up to one mismatch (MM) in the middle of the site. We divided the centered sites into four classes (WC 0MM, WC 1MM, GU 0MM, GU 1MM) for all analyses except those of the combined target sets of isomiRs and their canonical partners, in which we pooled all classes together.

### Statistical analysis of predicted binding sites and enrichment status

A FET was applied to ask whether transcripts containing at least one predicted binding site of a particular type were more likely than those with no site to be unambiguously classified as targets in the pull-down.

To investigate whether the location of the putative miRNA binding sites affected enrichment status, the following logistic regression model was fitted on the combined data from all pull-downs:

$$\begin{array}{l} \mathrm{logit}\left(p_{i,m}\right)=\ln\left(\frac{p_{i,m}}{1-p_{i,m}}\right)\\ \;\;=\beta_{0}+\beta_{1,1}x_{1,1,m.i}+\beta_{2,1}x_{2,1,m.i}+\ldots \\ \;\;+\beta_{j,k}x_{j,k,m.i} \end{array}$$

where *p*_*i,m*_ is the probability that the probe detecting transcript *i* is significantly enriched in the pull-down of miRNA *m*; *x*_*a,b,m,i*_ is the number of miR-*m* sites of type *a* in region *b* of transcript *i* (for example, miR-17-5p seed sites in the 3′ UTR); *β*_*a,b*_ is the coefficient (or weight) corresponding to that count and *β*_*0*_ is some constant. A value of *β*_*a,b*_ significantly greater than zero would indicate that, as the number of sites of type *a* in region *b* of a transcript increases, so too does the probability that the transcript is detected as significantly enriched in the pull-down. All coding transcripts with unambiguous enrichment status were included.

### Luciferase assays

Luciferase assays were carried out as previously described [8]. Briefly, complementary oligonucleotides corresponding to 54 nucleotides surrounding the miRNA binding site were annealed before being cloned into the *Spe*I and *Hin*dIII sites of pMIR-REPORT Luciferase (Ambion). A list of all oligonucleotides used is available in Table S6 in Additional file 1. All constructs were verified by capillary sequencing. HEK293T cells were co-transfected with 50 ng of a pMIR-REPORT Luciferase construct, 50 ng of pMIR-REPORT β-galactosidase (Ambion) and 10 nM of the appropriate mirVana miRNA mimic (Ambion/Life Technologies). After transfection, cells were incubated for 48 hours prior to harvesting. Luciferase activity was assayed using the Luciferase Assay System (Promega, Sydney, NSW, Australia), and detected on a Wallac 1420 luminometer (Perkin Elmer, Waltham, MA, USA). Transfection efficiency was assessed by β-galactosidase activity, using the β-Galactosidase Enzyme Assay System (Promega, Sydney, NSW, Australia), and detected on a PowerWave XS spectrophotometer (BioTek, Winooski, VT, USA).

### Quantitative RT-PCR

Primers used to determine whether transcripts were degraded after miRNA transfection are listed in Table S7 in Additional file 1. Cells were either mock-transfected, or transfected with biotinylated miRNAs as described in the Materials and methods section. RNA was purified from cell pellets using an RNeasy Mini Kit (QIAGEN). RNA integrity was assessed using an Agilent Bioanalyzer 2100, cDNA synthesis was carried out using random hexamer primers and SuperScript III (Invitrogen). qRT-PCR was performed using SYBR green PCR master-mix (Applied Biosystems) on an Applied Biosystems ViiA™ 7 Real time PCR system. Expression levels were calculated relative to those in the mock-transfected cells.

### Bio-layer interferometry

The generation and validation of the MDA-MB-231 miR-182-5p inducible cell line has been previously described [36]. miRNA expression was induced by the addition of 1 μg/ml doxycycline. Biotin-labeled miRNA duplexes were transfected into MDA-MB-231 cells using Dharmafect 4 (Thermo Fischer, Melbourne, VIC, Australia) according to the manufacturer’s instructions for this cell line. All cells were incubated in 5% CO_2_ for 48 hours prior to harvesting. RISC affinity purification was performed as previously described [46] with the following modifications: (i) the lysis buffer used was 10 mM Tris pH7.5 (Ambion), 10 mM KCl (Ambion), 1.5 mM MgCl_2_ (Ambion), 0.5 mM DTT (Invitrogen), 0.5% NP40 (Sigma Aldrich, Sydney, NSW, Australia), and 1X EDTA-free Complete protease inhibitor (Roche); (ii) cell lysates were cleared by centrifugation at 16,000 g for 30 minutes at 4°C. The miR-182-5p capture oligo and all binding site oligo sequences are shown in Table S8 in Additional file 1. Association and disassociation profiles were generated using the Octet Red (Forte Bio, Menlo Park, CA, USA) and streptavidin biosensors (Forte Bio) using the following parameters: lysis buffer for 60 seconds; RNA oligo for 10 minutes; saturated biotin (Sigma Aldrich) solution for 10 minutes; lysis buffer for 60 seconds; RISC solution for 10 minutes (association); lysis buffer for 10 minutes (disassociation). Wells were kept at 30°C, and rotated at 1,000 rpm to ensure a homogenous mix.

### Accession numbers

Data used in this manuscript are available from the Gene Expression Omnibus (GEO) under accession numbers GSE29101 (miR-10 family and mock transfection microarray data), GSE38593 (miR-182 microarray data), GSE40406 (miR-424 and miR-199a microarray data), and GSE55059 (all remaining microarray and sequencing data).

## Abbreviations

BLI: bio-layer interferometry; bp: base pair; CDS: coding sequence; ChIP: chromatin immunoprecipitation; CI: confidence interval; CLIP: crosslinking immunoprecipitation; FDR: false discovery rate; FET: Fisher’s exact test; HITS-CLIP: high-throughput sequencing of RNAs isolated by crosslinking immunoprecipitation; miRNA: microRNA; OR: odds ratio; PAR-CLIP: photoactivatable-ribonucleoside-enhanced crosslinking and immunoprecipitation; PCR: polymerase chain reaction; qRT-PCR: quantitative RT-PCR; RISC: RNA-induced silencing complex; UTR: untranslated region.

## Competing interests

This work was supported by Australian Research Council (ARC) Discovery Project Grants DP1093164 and DP0988754. NC is supported by an ARC Future Fellowship (FT120100453), and SMG is supported by a National Health and Medical Research Council (NHMRC) Senior Research Fellowship. AV is employed by a commercial company that supplies miRNA research and sequencing products. The funders had no role in study design, data collection and analysis, decision to publish, or preparation of the manuscript.

## Authors’ contributions

HCM analyzed the data and wrote the manuscript together with NC, who designed and supervised the study and conducted some of the experiments. SMG contributed to study design, supervision, and manuscript preparation. SW conducted the biotin pull-downs and AGO immuno-precipitations. ALS and KK performed the luciferase assays. KN ran the microarrays. EN performed the sequencing, and AV provided materials and assisted in experimental design. All authors read and approved the final manuscript.

## Supplementary Material

Additional file 1: Table S1 Sequences of the biotinylated miRNA duplexes. **Table S2.** miRNA-target interactions demonstrated by reporter assay [8,17,47-112]. **Table S3.** Results from Fisher’s exact tests for over-representation of genes implicated as targets via miRNA over-expression experiments, PAR-CLIP or luciferase assays amongst the set of genes significantly enriched in the pull-downs (5% FDR). **Table S4.** Results from Fisher’s exact tests examining enrichment of transcripts with a certain site type amongst transcripts targeted by two related miRNAs. **Table S5.** Summary of analysis of published studies. **Table S6.** Primers used for construction of pMIR-REPORT luciferase assay constructs. **Table S7.** Primers used for qRT-PCR analysis of mRNAs after transient transfection with biotinylated miRNA-duplexes. **Table S8.** Oligos used for RISC affinity purification and bio-layer interferometry. Click here for file

Additional file 2: Figure S1 Biotin pull-downs enrich for predicted and previously validated targets of miRNAs. **(A)** Left: two different miRNA duplexes, hsa-miR-424-3p (blue) and hsa-miR-199b-5p (red), were transfected into HEK293T cells, independently replicated three times. Total RNA samples (dotted lines) and pull-down miRNA enrichments (solid lines) were assayed by microarray, and clustered using the plotSampleRelations function of lumi. There is a very close relationship between the total RNA samples, even though they have been transfected with two different miRNAs with very different targets. Right: correlation of HEK293T control RNA transfected with either miR-424-3p or miR-199b-5p miRNAs. This demonstrates that there is very little effect of either duplex in this cell line, and that there is no major disruption of the underlying genetic networks upon transfection at this concentration. **(B,C)** Volcano plots showing the significance of the difference in expression between the indicated pull-down and the mock-transfected control, for all transcripts expressed in control cells. Targets predicted by TargetScan or validated previously via luciferase assay are indicated by orange and green dots, respectively. (B) A comparison between miR-10a biotin pull-down in this study (left) and by Ørom *et al.*[17] (right). *P*-values for the enrichment of luciferase validated targets or TargetScan predicted targets are indicated. Red dashed lines indicated the significance threshold and the fold-change threshold used in this study. For the pull-downs performed by Ørom *et al*., no enrichment of known or predicted targets was observed. (C) Volcano plots for all 10 miRNAs/isomiRs used in this study. Click here for file

Additional file 3: Figure S2 Distribution of log2 fold-change values for the miRNAs/isomiRs used in this study. Canonical miRNAs are plotted as solid lines. IsomiRs are plotted as dashed lines. Click here for file

Additional file 4: Figure S3 Targets identified through PAR-CLIP show greater enrichment in the biotin pull-down. Cumulative distribution of log fold-change in the biotin pull-down for transcripts identified as targets via PAR-CLIP [12] or not. Red, canonical transcripts containing at least one CLIP cluster (Table S5 in Additional file 1); black, all other canonical transcripts; *p*, one-sided *P*-value from Kolmogorov-Smirnov test for a difference in distributions. Click here for file

Additional file 5: Figure S4 RT-PCR of genes previously confirmed by luciferase assay. The bar plot indicates the expression of the indicated gene in HEK293T cells transfected with miR-17-5p relative to mock-transfected cells. These genes had previously been shown to be targeted by miR-17-5p in luciferase assays. Error bars indicate 95% confidence intervals calculated over three biological replicates. Asterisks indicate significantly reduced expression compared to mock-transfected cells (one-sided *t*-test). Click here for file

Additional file 6: Figure S5 Distribution of binding sites in miRNA transcripts. Frequency histograms showing the distribution of the number of each site type per transcript, for each of the 10 miRNAs used in this study (Table 1). Click here for file

Additional file 7: Figure S6 Proportion of transcripts with miRNA binding sites. Proportion of transcripts with a predicted binding site for the biotinylated miRNA in each pull-down. SE, significantly enriched in pull-down (5% FDR). Click here for file

Additional file 8: Figure S7 Effect of site location on enrichment in the biotin pull-down. **(A)** The odds ratios from Fisher’s exact tests for an enrichment of transcripts with the indicated site type amongst the putative target set including all transcripts. **(B,C)** Bar plots show the coefficient estimates from a logistic regression of enrichment status on site density (C) or site count (B). Only canonical transcripts were included. Error bars indicate 95% confidence intervals. Click here for file

Additional file 9: Figure S8 Hierarchical clustering of microarray data. Clustering was performed using the plotSampleRelations function in the *lumi* package. Total vertical distance between samples indicates similarity. Arrays A and B were Illumina HT-12 version 4 arrays, and array C was version 3. The miR-10b pull-down sample that clustered on a different branch to the other miR-10a and miR-10b samples (miR-10b_arrayA_1: red circle) was excluded. Click here for file
