# Supplementary material for: Imperfect centered miRNA binding sites are common and can mediate repression of target mRNAs
Source: Genome Biol. 2014 Mar 14;15(3):R51. doi: 10.1186/gb-2014-15-3-r51 (PMC4053950; doi:10.1186/gb-2014-15-3-r51)
Supplement: Additional file 1: Table S1 — Sequences of the biotinylated miRNA duplexes. Table S2. miRNA-target interactions demonstrated by reporter assay [8,17,47-112]. Table S3. Results from Fisher’s exact tests for over-representation of genes implicated as targets via miRNA over-expression experiments, PAR-CLIP or luciferase assays amongst the set of genes significantly enriched in the pull-downs (5% FDR). Table S4. Results from Fisher’s exact tests examining enrichment of transcripts with a certain site type amongst transcripts targeted by two related miRNAs. Table S5. Summary of analysis of published studies. Table S6. Primers used for construction of pMIR-REPORT luciferase assay constructs. Table S7. Primers used for qRT-PCR analysis of mRNAs after transient transfection with biotinylated miRNA-duplexes. Table S8. Oligos used for RISC affinity purification and bio-layer interferometry. [file gb-2014-15-3-r51-S1.doc]

**Supplementary Table 1.** Sequences of the biotinylated miRNA duplexes. A mismatch (indicated in bold) was incorporated into the reverse strand at position 2 so that the forward strand would be preferentially incorporated into RISC. B: biotin with a C6 spacer arm.

| **miRNA** | **Forward Strand** | **Reverse Strand** |
| --- | --- | --- |
| *miR-17-5p* | 5’-C**A**AAGUGCUUACAGUGCAGGUAG-B-3’ | 5’-ACCUGCACUGUAAGCACUU**A**GAG-3’ |
| *miR-17-5p-iso* | 5’-U**C**AAAGUGCUUACAGUGCAGGUA-B-3’ | 5’-CCUGCACUGUAAGCACUUU**A**AAG-3’ |
| *miR-182* | 5’-U**U**UGGCAAUGGUAGAACUCACACU-B-3’ | 5’-UGUGAGUUCUACCAUUGCCAU**A**AG-3’ |
| *miR-182-iso* | 5’-U**G**GCAAUGGUAGAACUCACACU-B-3’ | 5’-UGUGAGUUCUACCAUUGC**G**AAG-3’ |
| *miR-10a* | 5’-U**A**CCCUGUAGAUCCGAAUUUGUG-B-3’ | 5’-CAAAUUGGGAUCUACAGGG**C**AAG-3’ |
| *miR-10a-iso* | 5’-A**C**CCUGUAGAUCCGAAUUUGUGU-B-3’ | 5’-ACAAAUUGGGAUCUACAGG**A**UAG-3’ |
| *miR-10b* | 5’-U**A**CCCUGUAGAACCGAAUUUGUG-B-3’ | 5’-CAAAUUGGGUUCUACAGGG**C**AAG-3’ |
| *miR-10b-iso* | 5’-A**C**CCUGUAGAACCGAAUUUGUGU-B-3’ | 5’-ACAAAUUGGGUUCUACAGG**A**UAG-3’ |
| *miR-27a* | 5’-U**U**CACAGUGGCUAAGUUCCGC-B-3’ | 5’-GGAACUUAGCCACUGUG**C**AAG-3’ |
| *miR-23b* | 5’-A**U**CACAUUGCCAGGGAUUACC-B-3’ | 5’-UAAUCCCUGGCAAUGUG**C**UAG-3’ |

**Supplementary Table 2.** MiRNA-target interactions demonstrated by reporter assay. The genes targeted by the specified miRNAs are identified by their HUGO gene symbol. "Reference": the published study describing the interaction. "Expressed": whether or not the genes were expressed above background in HEK293T cells. "Pull-down": whether or not the interaction was observed in the corresponding biotinylated miRNA pull-down in this study, specifically whether the gene was detected by an Illumina probe that was significantly enriched in the pull-down. "Cell type": the cell line that was used in the original published study. For this study, all pull-downs were conducted in HEK293T cells.

| **miRNA** | **Gene** | **Reference** | **Expressed** | **Pull-down** | **Cell type** |
| --- | --- | --- | --- | --- | --- |
| *miR-10a* | USF2 | Agirre *et al.*, 2008 [47] | Yes | yes | RKO |
|  | BCL6 | Takahashi *et al*., 2012 [48] | No | - | CD4+ T cells |
|  | NCOR2 |  | Yes | No^b^ | CD4+ T cells |
|  | NCOR2 | Foley et al., 2011 [49] | Yes | No^b^ | SK-N-BE |
|  | IL12B | Xue *et al*., 2012 [50] | No | - | Dendritic cells |
|  | BTRC | Fang *et al.*, 2010 [51] | Yes | Yes | HEK293 |
|  | MAP3K7 |  | Yes | Yes^a^ | HEK293 |
|  | TRA2B | Meseguer et al., 2011 [52] | Yes | Yes | HeLa |
|  | SRSF1 |  | No | - | HeLa |
|  | PEBP1 | Orom *et al.*, 2008 [17] | Yes | No | HEK293 |
|  | RAN |  | Yes | Yes | HEK293 |
|  | HOXD4 | Tan *et al.*, 2009 [53] | No | - | MCF7 |
|  | HOXD10 |  | Yes | No | MCF7 |
|  | HOXA1 | Trakooljul *et al*., 2012 [54] | No | - | DF1 |
|  | ITGB |  | No | - | DF1 |
|  | ACSBG2 |  | No | - | DF1 |
|  | KLF11 |  | Yes | yes | DF1 |
|  | WDR11 |  | No | - | DF1 |
|  | NDRG1 |  | Yes | no | DF1 |
|  | FNBP1L |  | Yes | yes | DF1 |
|  | ACTG1 |  | Yes | no | DF1 |
|  | RHOB |  | Yes | no | DF1 |
|  | RHOBTB2 |  | Yes | no | DF1 |
|  | PDPK1 |  | Yes | Yes^a^ | DF1 |
|  | SDF2L1 |  | Yes | Yes | DF1 |
|  | CKAP4 |  | No | - | DF1 |
|  | PFKM |  | Yes | No | DF1 |
|  | MDM4 | Ovcharenko et al., 2011 [55] | No | - | HeLa |
| *miR-10b* | HOXD10 | Ma *et al.*, 2007 [56] | Yes | No | MDA-MB-231 |
|  | TRA2B | Meseguer et al., 2011 [52] | No | - | HeLa |
|  | SRSF1 |  | No | - | HeLa |
|  | TFAP2C | Gabriely et al., 2011 [57] | Yes | Yes | A172 |
| **miRNA** | **Gene** | **Reference** | **Expressed** | **Pull-down** | **Cell type** |
| *miR-10b* | BCL2L11 | Gabriely et al., 2011 [57] | Yes | No^b^ | A172 |
|  | CDKN1A |  | Yes | No | A172 |
|  | CDKN2A |  | Yes | No | HEK293 |
|  | SDC1 | Ibrahim *et al*., 2012 [58] | Yes | Yes | MDA-MB-231 and MCF7 |
|  | NCOR2 | Foley *et al*., 2011 [49] | Yes | No | SK-N-BE |
|  | BCL2L11 | Nishida *et al*., 2012 [59] | Yes | No^b^ | HCT-116 |
|  | TIAM1 | Moriarty *et al.*, 2010 [60] | Yes | No | SUM159PT |
|  | KLF4 | Tian *et al.*, 2010 [61] | Yes | No | KYSE140 and KYSE450 |
|  | CADM1 | Li et al., 2012 [62] | Yes | No | HepG2 |
| *miR-17-5p* | BMPR2 | Brock *et al.*, 2009 [63] | Yes | Yes | HEK293 |
|  | BCL2L11 | Cloonan *et al*., 2008 [8] | Yes | No | HEK293T |
|  | CDKN1A |  | Yes | Yes^b^ | HEK293T |
|  | CRK |  | Yes | No | HEK293T |
|  | E2F1 |  | Yes | No | HEK293T |
|  | HIF1A |  | Yes | Yes^a,b^ | HEK293T |
|  | MAPK9 |  | Yes | No | HEK293T |
|  | NCOA3 |  | Yes | Yes^b^ | HEK293T |
|  | PKD2 |  | Yes | No | HEK293T |
|  | PTEN |  | Yes | Yes^a^ | HEK293T |
|  | RBL1 |  | Yes | No | HEK293T |
|  | RBL2 |  | Yes | No^b^ | HEK293T |
|  | STAT3 |  | Yes | Yes | HEK293T |
|  | TP53INP1 |  | Yes | Yes | HEK293T |
|  | TSG101 |  | Yes | No | HEK293T |
|  | TGFBR2 | Dews *et al.*, 2010 [64] | Yes | Yes | DLD1 Dicer^hypo^ |
|  | CDKN1A | Fontana *et al.*, 2008 [65] | Yes | Yes^b^ | SH-EP |
|  | APP | Hebert *et al.*, 2009 [66] | Yes | No | HeLa |
|  | NCOA3 | Hossain *et al.*, 2006 [67] | Yes | Yes^b^ | HeLa |
|  | HBP1 | Li *et al.*, 2011 [68] | Yes | No | MDA-MB-231 |
|  | RBL2 | Lu *et al.*, 2007 [69] | Yes | No^b^ | HeLa |
|  | CCND1 | Yu *et al.*, 2008 [70] | Yes | No | MCF7 |
|  | HIF1A | Lichner *et al*., 2012 [71] | Yes | Yes^a,b^ | SKOV-3 |
|  | VHL |  | Yes | Yes^a^ | SKOV-3 |
|  | ZFPM2 | Xiang *et al*., 2012 [72] | Yes | No | HEK293T |
|  | CAPRIN2 | Riley *et al*., 2012 [73] | Yes | No | HEK293T |
|  | ZBTB4 | Kim et al., 2012 [74] | Yes | No | MDA-MB-231 and MCF7 |
| *miR-182* | ADCY6 | Saus *et al.*, 2010 [75] | Yes | Yes | HeLa |
|  | CLOCK |  | Yes | Yes | HeLa |
|  | TSC22 |  | No | - | HeLa |
|  | FOXO3 | Segura *et al.*, 2009 [76] | Yes | Yes | HEK293T |
|  | MITF |  | No | - | HEK293T |
|  | FOXO1 | Stittrich *et al.*, 2010 [77] | yes | Yes | CD4+ helper T cells |
|  | RGS17 | Sun *et al.*, 2010 [78] | yes | Yes | HEK293T |
|  | CTTN |  | yes | No | HEK293T |
|  | BRCA1 | Moskwa et al., 2011 [79] | yes | Yes | MDA-MB-231 and MCF7 |
|  | CREB1 | Kong et al., 2012 [80] | yes | No | MGC-803 |
|  | MTSS1 | Wang et al., 2012 [81] | yes | No | HUH-1 |
|  | SLC39A1 | Mihelich et al., 2011 [82] | yes | No | PrE |
| **miRNA** | **Gene** | **Reference** | **Expressed** | **Pull-down** | **Cell type** |
| *miR-182* | SOX2 | Weston et al., 2011 [83] | yes | No | HEK293 |
| *miR-23b* | GLS | Gao *et al.*, 2009 [84] | yes | No | MCF7 |
|  | HES1 | Kimura *et al.*, 2004 [85] | yes | No | P19 |
|  | SMAD3 | Rogler *et al.*, 2009 [86] | yes | Yes | HEK293 |
|  | SMAD4 |  | yes | No | HEK293 |
|  | SMAD5 |  | yes | Yes | HEK293 |
|  | MET | Salvi *et al.*, 2009 [87] | yes | Yes^b^ | SKHep1C3 |
|  | PLAU |  | yes | No^b^ | SKHep1C3 |
|  | TAB2 | Zhu et al., 2012 [88] | no | - | HEK293T |
|  | TAB3 |  | no | - | HEK293T |
|  | CHUK |  | yes | Yes | HEK293T |
|  | ADAMTS | Zhang *et al.*, 2011 [89] | no | - | HCT-116 |
|  | CNN2 |  | yes | No | HCT-116 |
|  | FZD7 |  | yes | No | HCT-116 |
|  | MAP3K1 |  | yes | Yes | HCT-116 |
|  | PAK2 |  | yes | Yes | HCT-116 |
|  | RRAS2 |  | yes | No | HCT-116 |
|  | TGFBR2 |  | yes | No | HCT-116 |
|  | PLAU |  | yes | No^b^ | HCT-116 |
|  | ERBB4 |  | no | - | HCT-116 |
|  | HOXA3 |  | yes | No | HCT-116 |
|  | LAMP1 |  | no | - | HCT-116 |
|  | LPP |  | yes | Yes | HCT-116 |
|  | MEF2C |  | no | - | HCT-116 |
|  | MET |  | yes | Yes^b^ | HCT-116 |
|  | PRDM1 |  | no | - | HCT-116 |
|  | REPS2 |  | no | - | HCT-116 |
|  | SEMA6D |  | no | -^b^ | HCT-116 |
|  | YES1 |  | yes | Yes | HCT-116 |
|  | VHL | Chen *et al.*, 2012 [90] | yes | Yes | U87 and LN229 |
|  | PRDX3 | He *et al.*, 2012 [91] | yes | No | DU145 |
|  | PTEN | Nicholls *et al.*, 2011 [92] | yes | Yes | HEK293T |
|  | ETS15 |  | no | - | HEK293T |
|  | PRKACB | Ham *et al.*, 2012 [93] | no | - | hMSCs |
|  | SPRY2 | Zhou *et al.*, 2011 [94] | yes | yes | HUVEC |
|  | SEMA6A |  | yes | No | HUVEC |
|  | SEMA6D |  | no | -^b^ | HUVEC |
|  | LRAT | Amann et al., 2012 [95] | no | - | HEK293T and MeWo |
|  | FBXO32 | Wada et al., 2011 [96] | yes | No | HeLa |
|  | TRIM63 |  | no | - | HeLa |
| *miR-27a* | PPARG | Kim *et al.*, 2010 [97] | yes | Yes^a^ | HeLa |
|  | PHB | Liu *et al.*, 2009 [98] | yes | No | MGC803 |
|  | FBXW7 | Wang et al., 2011 [99] | yes | Yes^a^ | HBELH |
|  | ZBTB10 | Liu et al., 2012 [100] | no | - | BT474 and MDA-MB-453 |
|  | SATB2 | Hassan et al., 2010 [101] | yes | Yes | MC3T3-E1 |
|  | FOXO1 | Guttilla et al., 2009 [102] | yes | Yes | MCF7 |
|  | SPRY2 | Ma et al., 2010 [103] | yes | Yes | HEK293T and PANC-1 |
|  | SEMA6A | Urbich et al., 2012 [104] | yes | No | HUVEC |
| **miRNA** | **Gene** | **Reference** | **Expressed** | **Pull-down** | **Cell type** |
| miR-27a | MSTN | Huang et al., 2012 [105] | no | - | HEK293T |
|  | GTF2H2 | Portal et al., 2011 [106] | yes | No | HEK293 |
|  | THRB | Nishi et al., 2011 [107] | no | - | HEK293 |
|  | DPYD | Hirota et al., 2012 [108] | no | - | HepG2 |
|  | CXCL12 | Lu et al., 2012 [109] | no | - | HEK293 |
|  | FADD | Chhabra et al., 2009 [110] | yes | No | HEK293T |
|  | RXRA | Ji et al., 2009 [111] | yes | Yes | HEK293T |
|  | RUNX1 | Ben-ami et al., 2009 [112] | no | - | HEK293 |

^a^Detected by at least one Illumina probe that was significantly enriched and at least one Illumina probe that was not. These genes were classed as “ambiguous” in Supplementary Table 3.

^b^Although these genes are listed twice in this table (because they were verified in different studies), they were each only counted once in the Fisher’s Exact Test.

**Supplementary Table 3.** Results from Fisher’s Exact Tests for over-representation of genes implicated as targets *via* miRNA over-expression experiments, PAR-CLIP or luciferase assays amongst the set of genes significantly enriched in the pull-downs (5% FDR). Red text indicates that the p-value for that test did not reach statistical significance. OR refers to the odds ratio. 95% CI (L) and 95% CI (U) refer to the lower and upper ranges of the 95% confidence interval of the odds ratio, respectively.

| **method** | **miRNA** | **FET results (ambiguous excluded)** | | | | **FET results (ambiguous counted)** | | | | **target in other dataset** | | | **not target in other dataset** | | |
| --- | --- | --- | --- | --- | --- | --- | --- | --- | --- | --- | --- | --- | --- | --- | --- |
|  |  | **OR** | **95% CI (L)** | **95% CI (U)** | **one-sided p-value** | **OR** | **95% CI (L)** | **95% CI (U)** | **one-sided p-value** | **pull-down target** | **not pull-down target** | **Ambig-uous** | **pull-down target** | **not pull-down target** | **ambiguous** |
| luciferase assays | *miR-10a* | 5.90 | 2.1 | 16.3 | 1.52E-03 | 5.92 | 2.3 | 15.4 | 4.91E-04 | 7 | 8 | 2 | 1085 | 7317 | 306 |
|  | *miR-10b* | 1.36 | 0.3 | 6.6 | 4.80E-01 | 1.10 | 0.2 | 5.3 | 5.83E-01 | 2 | 7 | 0 | 1451 | 6919 | 346 |
|  | *miR-17-5p* | 5.16 | 2.0 | 13.5 | 3.13E-03 | 5.97 | 2.6 | 13.8 | 1.84E-04 | 6 | 14 | 3 | 652 | 7856 | 194 |
|  | *miR-182* | 8.59 | 2.6 | 28.2 | 9.12E-04 | 6.88 | 2.1 | 22.6 | 2.55E-03 | 6 | 5 | 0 | 1036 | 7421 | 258 |
|  | *miR-23b* | 5.94 | 2.6 | 13.5 | 7.19E-05 | 4.79 | 2.1 | 10.9 | 3.86E-04 | 11 | 12 | 0 | 1127 | 7303 | 272 |
|  | *miR-27a* | 8.90 | 2.2 | 35.7 | 5.26E-03 | 10.05 | 2.8 | 35.7 | 6.38E-04 | 4 | 4 | 2 | 852 | 7584 | 280 |
|  | ***combined*** | 5.15 | 3.4 | 7.9 | 6.18E-12 | 4.86 | 3.2 | 7.3 | 1.01E-12 | 36 | 50 | 7 | 6203 | 44400 | 1656 |
| TargetScan predictions | *miR-10a* | 3.53 | 2.6 | 4.9 | 1.51E-13 | 3.21 | 2.4 | 4.4 | 1.34E-12 | 68 | 93 | 6 | 1463 | 7071 | 290 |
|  | *miR-10a-iso* | 1.53 | 1.0 | 2.4 | 3.49E-02 | 1.54 | 1.0 | 2.3 | 1.64E-02 | 32 | 63 | 6 | 2113 | 6380 | 390 |
|  | *miR-10b* | 3.02 | 2.2 | 4.2 | 1.99E-10 | 3.03 | 2.2 | 4.2 | 1.10E-11 | 66 | 81 | 13 | 1802 | 6677 | 345 |
|  | *miR-10b-iso* | 2.32 | 1.5 | 3.5 | 7.92E-05 | 1.87 | 1.2 | 2.8 | 2.42E-03 | 37 | 65 | 0 | 1668 | 6807 | 408 |
|  | *miR-17-5p* | 5.21 | 4.3 | 6.3 | 2.09E-56 | 4.65 | 3.9 | 5.5 | 8.76E-57 | 197 | 378 | 33 | 746 | 7463 | 231 |
|  | *miR-17-5p-iso* | 6.75 | 5.2 | 8.8 | 3.51E-42 | 6.23 | 4.9 | 8.0 | 9.57E-43 | 120 | 124 | 15 | 1067 | 7449 | 234 |
|  | *miR-182* | 2.18 | 1.8 | 2.6 | 2.41E-18 | 1.82 | 1.7 | 2.4 | 7.19E-17 | 229 | 451 | 26 | 1525 | 6540 | 295 |
|  | *miR-182-iso* | 5.35 | 3.9 | 7.3 | 5.63E-23 | 4.60 | 3.4 | 6.2 | 1.39E-20 | 75 | 93 | 5 | 1118 | 7425 | 271 |
|  | *miR-23b* | 2.77 | 2.4 | 3.3 | 7.05E-31 | 2.57 | 2.2 | 3.0 | 6.10E-30 | 251 | 447 | 39 | 1348 | 6656 | 333 |
|  | *miR-27a* | 3.07 | 2.6 | 3.6 | 2.40E-37 | 2.96 | 2.5 | 3.5 | 3.27E-40 | 266 | 412 | 55 | 1385 | 6581 | 349 |
|  | ***combined*** | 2.96 | 2.7 | 3.2 | 8.84E-180 | 2.78 | 2.6 | 3.0 | 7.76E-179 | 1341 | 2207 | 198 | 14235 | 69049 | 3146 |
| miRNA over-expression | *miR-17-5p* | 2.90 | 2.2 | 3.9 | 1.54E-11 | 2.47 | 1.9 | 3.2 | 6.99E-10 | 67 | 205 | 9 | 856 | 7588 | 282 |
|  | *miR-17-5p* | 2.87 | 2.0 | 4.1 | 6.09E-08 | 2.46 | 1.8 | 3.4 | 6.38E-07 | 43 | 133 | 6 | 856 | 7588 | 282 |
| PAR-CLIP | *miR-17-5p* | 3.05 | 2.2 | 3.4 | 1.27E-17 | 2.86 | 2.2 | 3.2 | 5.17E-20 | 131 | 524 | 31 | 673 | 7440 | 191 |
|  | *miR-182* | 1.30 | 1.1 | 1.6 | 8.26E-03 | 1.35 | 1.1 | 1.6 | 1.28E-03 | 154 | 505 | 31 | 1507 | 6538 | 255 |
|  | *miR-27a* | 2.48 | 1.9 | 3.1 | 1.56E-09 | 2.76 | 2.1 | 3.2 | 8.48E-15 | 107 | 227 | 34 | 1335 | 6967 | 320 |
|  | *miR-23b* | 1.72 | 1.4 | 2.5 | 2.24E-03 | 2.11 | 1.6 | 2.8 | 1.53E-06 | 66 | 179 | 24 | 1363 | 7060 | 298 |
|  | *miR-10a* | 1.30 | 0.9 | 2.0 | 1.54E-01 | 1.44 | 1.0 | 2.1 | 4.68E-02 | 33 | 117 | 9 | 1448 | 7105 | 278 |
|  | *miR-10b* | 1.97 | 1.3 | 3.3 | 1.30E-02 | 2.15 | 1.4 | 3.3 | 2.02E-03 | 23 | 85 | 6 | 1013 | 7667 | 196 |
|  | *miR-17-5p-iso* | 2.40 | 1.9 | 3.2 | 4.05E-08 | 2.67 | 2.1 | 3.4 | 2.89E-12 | 85 | 254 | 24 | 1000 | 7427 | 200 |
|  | *miR-182-iso* | 1.37 | 1.1 | 1.8 | 2.83E-02 | 1.44 | 1.1 | 1.8 | 5.36E-03 | 71 | 351 | 19 | 1044 | 7266 | 239 |
|  | *miR-10a-iso* | 1.70 | 1.2 | 2.4 | 9.31E-03 | 1.76 | 1.3 | 2.4 | 2.19E-03 | 39 | 163 | 10 | 1051 | 7483 | 244 |
|  | *miR-10b-iso* | 1.55 | 1.2 | 3.2 | 1.20E-01 | 2.03 | 1.5 | 3.4 | 5.98E-03 | 19 | 128 | 8 | 617 | 8070 | 148 |
|  | ***combined*** | 1.85 | 1.7 | 2.1 | 3.94E-32 | 1.99 | 1.8 | 2.1 | 2.44E-50 | 728 | 2533 | 196 | 11051 | 73023 | 2369 |

**Supplementary Table 4.** Results from Fisher's exact tests examining enrichment of transcripts with a certain site type amongst transcripts targeted by two related miRNAs. These tests were based only on probes that bound to a single canonical transcript with a single site type. Group A contains transcripts significantly enriched in the pull-downs of both indicated miRNAs, whereas Group B contains those significant only in the pull-down of the single indicated miRNA. We expected to see a significant p-value in the cells shaded light grey. Thus, we would expect transcripts targeted by, for example, miR-10a and miR-10a-iso, to be depleted of seed sites (which differ between them) relative to transcripts targeted by only miR-10a or only miR-10a-iso. OR refers to the odds ratio. 95% CI (L) and 95% CI (U) refer to the lower and upper ranges of the 95% confidence interval of the odds ratio, respectively.

| **Group A** | **Group B** | **Site** | **Upper-tailed**  **p-value** | **Lower-tailed**  **p-value** | **OR** | **95% CI (L)** | **95% CI (U)** | **Confirms expectations** |
| --- | --- | --- | --- | --- | --- | --- | --- | --- |
| *miR-10a*  *vs*  *miR-10b* | 10a | seed | 2.02E-03 | 9.99E-01 | 2.14 | 1.28 | 3.58 | Yes |
|  | 10b | seed | 5.33E-32 | 1.00E+00 | 11.57 | 7.19 | 18.74 | Yes |
|  | 10a | centered | 9.94E-01 | 7.94E-03 | 0.75 | 0.59 | 0.94 | Yes |
|  | 10b | centered | 1.00E+00 | 4.68E-04 | 0.64 | 0.50 | 0.83 | Yes |
| *miR-10a*  *vs*  *miR-10a-iso* | 10a | seed | 2.07E-01 | 8.58E-01 | 1.27 | 0.77 | 2.11 | No |
|  | 10a-iso | seed | 9.90E-01 | 2.12E-02 | 0.53 | 0.29 | 0.95 | Yes |
|  | 10a | centered | 1.69E-02 | 9.88E-01 | 1.30 | 1.03 | 1.65 | Yes |
|  | 10a-iso | centered | 2.82E-06 | 1.00E+00 | 2.07 | 1.51 | 2.85 | Yes |
| *miR-10b*  *vs*  *miR-10b-iso* | 10b | seed | 4.28E-01 | 6.49E-01 | 1.06 | 0.71 | 1.57 | No |
|  | 10b-iso | seed | 9.98E-01 | 5.04E-03 | 0.49 | 0.29 | 0.84 | Yes |
|  | 10b | centered | 1.48E-03 | 9.99E-01 | 1.52 | 1.16 | 1.99 | Yes |
|  | 10b-iso | centered | 1.11E-03 | 9.99E-01 | 1.78 | 1.24 | 2.56 | Yes |
| *miR-182*  *vs*  *miR-182-iso* | 182 | seed | 6.05E-01 | 5.06E-01 | 0.97 | 0.56 | 1.68 | No |
|  | 182-iso | seed | 8.14E-01 | 2.63E-01 | 0.82 | 0.50 | 1.36 | No |
|  | 182 | centered | 1.87E-02 | 9.88E-01 | 1.45 | 1.04 | 2.04 | Yes |
|  | 182-iso | centered | 9.40E-07 | 1.00E+00 | 2.08 | 1.54 | 2.80 | Yes |
| *miR-17-5p*  *vs*  *miR-17-5p-iso* | 17-5p | seed | 9.96E-01 | 1.10E-02 | 0.40 | 0.19 | 0.84 | Yes |
|  | 17-5p-iso | seed | 2.12E-01 | 8.51E-01 | 1.25 | 0.78 | 2.02 | No |
|  | 17-5p | centered | 1.19E-03 | 9.99E-01 | 2.09 | 1.32 | 3.32 | Yes |
|  | 17-5p-iso | centered | 1.83E-01 | 8.61E-01 | 1.20 | 0.84 | 1.71 | No |

**Supplementary Table 5.** Summary of analysis of published studies.

| **miRNA** | **Method** | **Cell type** | **Platform** | **Reference** | **Data analysis** | **Criteria to consider a gene/transcript as a miRNA target** |
| --- | --- | --- | --- | --- | --- | --- |
| *all* | PAR-CLIP | HEK293 | high-throughput sequencing | Hafner *et al.* 2010 [11] | Obtained stringent CLIP clusters with biological complexity > 1 (a measure of reproducibility used by Chi *et al.* [16]) from starBase [59] ; searched for seed and centered sites within clusters that overlapped Ensembl transcripts | Canonical transcript contained a robust cluster (biological complexity > 1 and > 4 tags) that covered a predicted binding site for that miRNA |
| *miR-17-5p* | transfection with pre-miR-17-5p vs. mock-transfections | HUVECs | microarray (Affymetrix Human Genome U133 Plus 2.0 Array) | Doebele *et al.* 2010 [14] | Downloaded the RMA-normalized data from GEO (GSE20745)**;** did analysis of differential expression as described in Methods | HGNC gene detected by a probe with adjusted one-sided p-value < 0.15 (after multiple-testing correction, the lowest p-value for repression was 0.10) |
| *miR-17-5p* | transfection with pre-miR-17-5p vs. mock-transfections | HCT116 Dicer -/- cells | microarray (Rosetta/Merck Human 44k 1.1 microarray) | Ivanovska *et al.* 2008 [15] | Downloaded data from GEO (GSM371207); this had been pre-processed in Rosetta; retained probes with a quality score of 1; used p-value for differential expression generated by Rosetta | HGNC gene detected by a probe with a nominal two-sided p < 0.01 that was expressed more highly in controls |

**Supplementary Table 6.** Primers used for construction of pMIR-REPORT luciferase assay constructs.

| **miRNA** | **Gene Symbol** | **Primer Orientation** | **Primer Sequence (5’ -> 3’)** |
| --- | --- | --- | --- |
| miR-17-5p | MAPK3 | forward | CTAGACTACCTGGATCAGCTCAACCACATTCTGGGCATCCTGGGCTCCCCATCCCAGGAG |
| miR-17-5p | MAPK3 | reverse | AGCTCTCCTGGGATGGGGAGCCCAGGATGCCCAGAATGTGGTTGAGCTGATCCAGGTAGT |
| miR-17-5p | DULLARD | forward | CTAGAAGAGGAGATATTACAGACAGCACTGCACTTTGGAGTTGGGCAGCTACATCAAGGA |
| miR-17-5p | DULLARD | reverse | AGCTTCCTTGATGTAGCTGCCCAACTCCAAAGTGCAGTGCTGTCTGTAATATCTCCTCTT |
| miR-17-5p | RPIA | forward | CTAGTGATTTGTTTTTAGTTAGTTTTTATTGTGAGCACACATAGTACCTAGTTACATCTT |
| miR-17-5p | RPIA | reverse | AGCTAAGATGTAACTAGGTACTATGTGTGCTCACAATAAAAACTAACTAAAAACAAATCA |
| miR-17-5p | APH1A | forward | CTAGATGGGGGCTGCGGTGTTTTTCGGCTGCACTTTCGTCGCGTTCGGCCCGGCCTTCGC |
| miR-17-5p | APH1A | reverse | AGCTGCGAAGGCCGGGCCGAACGCGACGAAAGTGCAGCCGAAAAACACCGCAGCCCCCAT |
| miR-17-5p | RPP21 | forward | CTAGCAAATAAAGTTTACTTGTTTTACATTCCATGATTCTGTTCTGTGGGTATTTCAACT |
| miR-17-5p | RPP21 | reverse | AGCTAGTTGAAATACCCACAGAACAGAATCATGGAATGTAAAACAAGTAAACTTTATTTG |
| miR-17-5p | AKT1 | forward | CTAGGAGTACCTGAAGCTGCTGGGCAAGGGCACTTTCGGCAAGGTGATCCTGGTGAAGGA |
| miR-17-5p | AKT1 | reverse | AGCTTCCTTCACCAGGATCACCTTGCCGAAAGTGCCCTTGCCCAGCAGCTTCAGGTACTC |
| miR-17-5p | PPP2R2B | forward | CTAGCTTCCCCTTCGCCTTCTGCCATGATTGTAAGTTTCCTGAGACCTCCCCAGCCATGC |
| miR-17-5p | PPP2R2B | reverse | AGCTGCATGGCTGGGGAGGTCTCAGGAAACTTACAATCATGGCAGAAGGCGAAGGGGAAG |
| miR-17-5p | FOXA1 | forward | CTAGGTGTGCTTGTTTCATCCAGTGTTATGCACTTTCCACAGTTGGACATGGTGTTAGTA |
| miR-17-5p | FOXA1 | reverse | AGCTTACTAACACCATGTCCAACTGTGGAAAGTGCATAACACTGGATGAAACAAGCACAC |
| miR-17-5p | PRKAG1 | forward | CTAGCTTGCAACATCGATCACATTACTTTGAGGGTGTTCTCAAGTGCTACCTGCATGAGA |
| miR-17-5p | PRKAG1 | reverse | AGCTTCTCATGCAGGTAGCACTTGAGAACACCCTCAAAGTAATGTGATCGATGTTGCAAG |
| miR-17-5p | TSPAN4 | forward | CTAGTGTGGCTTCAGGAGAACCTGCTGGCTGTGGGCATCTTTGGGCTGTGCACGGCGCTG |
| miR-17-5p | TSPAN4 | reverse | AGCTCAGCGCCGTGCACAGCCCAAAGATGCCCACAGCCAGCAGGTTCTCCTGAAGCCACA |
| miR-17-5p | DYNLT1 | forward | CTAGCTTTTCTCATTCTCTTTGTTTTGTGGCACTTTCACAATGTAGAGGAAAAAACCAAA |
| miR-17-5p | DYNLT1 | reverse | AGCTTTTGGTTTTTTCCTCTACATTGTGAAAGTGCCACAAAACAAAGAGAATGAGAAAAG |
| miR-17-5p | SKP2 | forward | CTAGTCAAATTTAGTGCGACTTAACCTTTCTGGGTGTTCTGGATTCTCTGAATTTGCCCT |
| miR-17-5p | SKP2 | reverse | AGCTAGGGCAAATTCAGAGAATCCAGAACACCCAGAAAGGTTAAGTCGCACTAAATTTGA |
| miR-27a | PPP2R5C | forward | CTAGAACGTACTGGGCAAATGACAATCCTCAGCCGCTGGTATTTTCTAAGGGGTCTCTTC |
| miR-27a | PPP2R5C | reverse | AGCTGAAGAGACCCCTTAGAAAATACCAGCGGCTGAGGATTGTCATTTGCCCAGTACGTT |
| miR-27a | FYN | forward | CTAGACTTGTCCCCAAATCCGAACCTCCTCTGTGAAGCATTCGAGACAGAACCTTGTTAT |
| miR-27a | FYN | reverse | AGCTATAACAAGGTTCTGTCTCGAATGCTTCACAGAGGAGGTTCGGATTTGGGGACAAGT |
| miR-27a | MAPKAPK5 | forward | CTAGTAGGGTGCAGGACTTAATAATAGTATAGTTATTGTTTGTTTTTAAGAAAAGCTCAG |
| miR-27a | MAPKAPK5 | reverse | AGCTCTGAGCTTTTCTTAAAAACAAACAATAACTATACTATTATTAAGTCCTGCACCCTA |
| miR-27a | CNOT7 | forward | CTAGTTCTTATGACTTCTGGAGTGGTCCTCTGTGAAGGGGTCAAATGGTTGTCATTTCAT |
| **miRNA** | **Gene Symbol** | **Primer Orientation** | **Primer Sequence (5’ -> 3’)** |
| miR-27a | CNOT7 | reverse | AGCTATGAAATGACAACCATTTGACCCCTTCACAGAGGACCACTCCAGAAGTCATAAGAA |
| miR-27a | RHOA | forward | CTAGGTGGTTTCATGTTAGTTACCTTATAGTTACTGTGTAATTAGTGCCACTTAATGTAT |
| miR-27a | RHOA | reverse | AGCTATACATTAAGTGGCACTAATTACACAGTAACTATAAGGTAACTAACATGAAACCAC |
| miR-27a | LEF1 | forward | CTAGACAGCTTGTCTGGTAAGTGGCTTCTCTGTGAATTGCCTGTAACACATAGTGGCTTC |
| miR-27a | LEF1 | reverse | AGCTGAAGCCACTATGTGTTACAGGCAATTCACAGAGAAGCCACTTACCAGACAAGCTGT |
| miR-27a | MAP2K3 | forward | CTAGCATCCCGGGTCCTTTCCCTGATGGGTTGGGGCAGTTACCTGGTTGCTGTTTTAATT |
| miR-27a | MAP2K3 | reverse | AGCTAATTAAAACAGCAACCAGGTAACTGCCCCAACCCATCAGGGAAAGGACCCGGGATG |
| miR-27a | TGFBR2 | forward | CTAGGGGTCGGGGGCTGCTCAGGGGCCTGTGGCCGCTGCACATCGTCCTGTGGACGCGTA |
| miR-27a | TGFBR2 | reverse | AGCTTACGCGTCCACAGGACGATGTGCAGCGGCCACAGGCCCCTGAGCAGCCCCCGACCC |
| miR-27a | RALB | forward | CTAGGAAGTAGAGAGAGATAAGCCATCGCCCCTTTGCCTCTGAGAATTGGCTGCTGTTTC |
| miR-27a | RALB | reverse | AGCTGAAACAGCAGCCAATTCTCAGAGGCAAAGGGGCGATGGCTTATCTCTCTCTACTTC |
| miR-27a | PRKCI | forward | CTAGCAAGCCAAGCGTTTCAACAGGCGTGCTCACTGTGCCATCTGCACAGACCGAATATG |
| miR-27a | PRKCI | reverse | AGCTCATATTCGGTCTGTGCAGATGGCACAGTGAGCACGCCTGTTGAAACGCTTGGCTTG |
| miR-27a | E2F6 | forward | CTAGAGTTTACTCTGTAGTTTACCATTAGACTGTGAGCTCCTTGAGGGACTTTGTCATAA |
| miR-27a | E2F6 | reverse | AGCTTTATGACAAAGTCCCTCAAGGAGCTCACAGTCTAATGGTAAACTACAGAGTAAACT |
| miR-27a | PPARG | forward | CTAGAGGACTTGTACTAGCAGAGAGTCCTGAGCCACTGCCAACATTTCCCTTCTTCCAGT |
| miR-27a | PPARG | reverse | AGCTACTGGAAGAAGGGAAATGTTGGCAGTGGCTCAGGACTCTCTGCTAGTACAAGTCCT |
| miR-182 | MAX | forward | CTAGTGTGTGTGTGGGGGGGACTCGGCTTGTTGTTGTCGGTGACTTCCCCCTCCCCTTCA |
| miR-182 | MAX | reverse | AGCTTGAAGGGGAGGGGGAAGTCACCGACAACAACAAGCCGAGTCCCCCCCACACACACA |
| miR-182 | PRKAG1 | forward | CTAGACAGATTGGCACCTATGCCAATATTGCTATGGTTCGCACTACCACCCCCGTCTATG |
| miR-182 | PRKAG1 | reverse | AGCTCATAGACGGGGGTGGTAGTGCGAACCATAGCAATATTGGCATAGGTGCCAATCTGT |
| miR-182 | SMARCD3 | forward | CTAGAGATTCCCCAGCGCCTCACAGCCCTGCTATTGCCCCCTGACCCAATTGTCATCAAC |
| miR-182 | SMARCD3 | reverse | AGCTGTTGATGACAATTGGGTCAGGGGGCAATAGCAGGGCTGTGAGGCGCTGGGGAATCT |
| miR-182 | EIF4G2 | forward | CTAGTCGTTCCTAATGAATAAAAATCAAGTGCCAAAGCTTCAGCCCCAGATAACTATGAT |
| miR-182 | EIF4G2 | reverse | AGCTATCATAGTTATCTGGGGCTGAAGCTTTGGCACTTGATTTTTATTCATTAGGAACGA |
| miR-182 | CASP7 | forward | CTAGTGTTTTGGCTTTATGTGCAAAATCTGTTATAGCTTTAAAATATATCTGGAACTTTT |
| miR-182 | CASP7 | reverse | AGCTAAAAGTTCCAGATATATTTTAAAGCTATAACAGATTTTGCACATAAAGCCAAAACA |
| miR-182 | PRKCD | forward | CTAGAGTGTCTGAGGTGACCGTGGGTGTGTCGGTGCTGGCCGAGCGCTGCAAGAAGAACA |
| miR-182 | PRKCD | reverse | AGCTTGTTCTTCTTGCAGCGCTCGGCCAGCACCGACACACCCACGGTCACCTCAGACACT |
| miR-182 | FKBP1A | forward | CTAGAGCACCATTTATGAGTCTCAAGTTTTATTATTGCAATAAAAGTGCTTTATGCCGGC |
| miR-182 | FKBP1A | reverse | AGCTGCCGGCATAAAGCACTTTTATTGCAATAATAAAACTTGAGACTCATAAATGGTGCT |
| miR-182 | CHEK2 | forward | CTAGATATCCAGCTCCTCTACCAGCACGATGCCAAACTCCAGCCAGTCCTCTCACTCCAG |
| miR-182 | CHEK2 | reverse | AGCTCTGGAGTGAGAGGACTGGCTGGAGTTTGGCATCGTGCTGGTAGAGGAGCTGGATAT |
| miR-182 | PDPK1 | forward | CTAGAGAAGCTGTATTTCGGCCTTAGTTATGCCAAAAATGGAGAACTACTTAAATATATT |
| **miRNA** | **Gene Symbol** | **Primer Orientation** | **Primer Sequence (5’ -> 3’)** |
| miR-182 | PDPK1 | reverse | AGCTAATATATTTAAGTAGTTCTCCATTTTTGGCATAACTAAGGCCGAAATACAGCTTCT |
| miR-182 | CDKN1B | forward | CTAGTTTGTAATGTGTGAAAAAGATGCCAATTATTGTTACACATTAAGTAATCAATAAAG |
| miR-182 | CDKN1B | reverse | AGCTCTTTATTGATTACTTAATGTGTAACAATAATTGGCATCTTTTTCACACATTACAAA |
| miR-182 | RBPJ | forward | CTAGGGAGTTGAAAAATGGAAGAATTATTTGCCAAAAGAGGAGGACAAAAGATAATATGC |
| miR-182 | RBPJ | reverse | AGCTGCATATTATCTTTTGTCCTCCTCTTTTGGCAAATAATTCTTCCATTTTTCAACTCC |
| miR-182 | PRKAG2 | forward | CTAGAAGCCCTGATCCTCACACCAGCAGGTGCCAAACAAAAGGAGACAGAAACGGAGTGA |
| miR-182 | PRKAG2 | reverse | AGCTTCACTCCGTTTCTGTCTCCTTTTGTTTGGCACCTGCTGGTGTGAGGATCAGGGCTT |
| miR-182 | NFKBIB | forward | CTAGCGACCCCCGCCCCGTGTGATTTGTTTCATTGTTAATATAATTTCCAGTTTAATAAA |
| miR-182 | NFKBIB | reverse | AGCTTTTATTAAACTGGAAATTATATTAACAATGAAACAAATCACACGGGGCGGGGGTCG |

**Supplementary Table 7.** Primers used for qRT-PCR analysis of mRNAs after transient transfection with biotinylated miRNA-duplexes.

| **Gene Symbol**  **(primer pair)** | **Forward primer** | **Reverse primer** |
| --- | --- | --- |
| RBL1 | GTAGCACTGGGGCTCAGGTA | GTACCACCGCCATTCAGAGT |
| PKD2 | TCCATGTGTATCGACCATGTG | TTTTGCAAGTGAAATGAAAAACA |
| MAPK9 | GCAGGCAATCCTATCAGGTC | CCTGAAATGCTTGGGAGTTG |
| TSG101 (A) | TTTGTGGTTTGCAAGGTCAG | GGCGTGATAGACCTGGATGT |
| TSG101 (B) | CTGTTTACGGGACAGAAGACG | GGCGTGATAGACCTGGATGT |
| HIF1A (A) | TGCCAGCTCAAAAGAAAACA | ACCAACAGGGTAGGCAGAAC |
| HIF1A (B) | CAGAAACCTACTGCAGGGTGA | GAGCCACCAGTGTCCAAAAA |
| KPNA2 (A) | CACAAAAGGAAGCTGTGTGG | CGGTTCTATTATGCCACAGTGA |
| KPNA2 (B) | TGTGGTTTGTTACTGTAGCACTTTT | CCTAGTCAGAGGACAAGCTTCA |
| KPNA2 (C) | CACAAAAGGAAGCTGTGTGG | CAGGATGCTGCAAGATAGGA |

**Supplementary Table 8.** Oligos used for RISC affinity purification and bio-layer interferometry.

| **Name** | **Sequence (5’ – 3’)** | **Comments** |
| --- | --- | --- |
| 182 capture | TCTTCCTGTGTCTGGAGCAACTTGCCAACACCTT | 2-O-me modified DNA, biotin labeled at 5’ end. |
| 182 competitor | AAGGTGTTGGCAAGTTGCTCCAGACACAGGAAGA | DNA |
| 182 seed | UCUUCCUGUGUCUGGAGCAACUUGCCAACACCUU | RNA, biotin labeled at 5’ end |
| 182 perfect | UCUUCCUGUGUCUGUCUACCAUUGCACCCACCUU | RNA, biotin labeled at 5’ end |
| 182 imperfect | UCUUCCUGUGUCUGUCUACAAUUGCACCCACCUU | RNA, biotin labeled at 5’ end |
| 182 imperfectGU | UCUUCCUGUGUCUGUUUGCAGUUGCACCCACCUU | RNA, biotin labeled at 5’ end |
| 182 3-prime | UCUUCCGUGUGAGGGAGCAACGGUAACCCACCUU | RNA, biotin labeled at 5’ end |
