# Supplementary figures and images for: Imperfect centered miRNA binding sites are common and can mediate repression of target mRNAs
Source: Genome Biol. 2014 Mar 14;15(3):R51. doi: 10.1186/gb-2014-15-3-r51 (PMC4053950; doi:10.1186/gb-2014-15-3-r51)

# SUPPLEMENTARY FIGURE 1

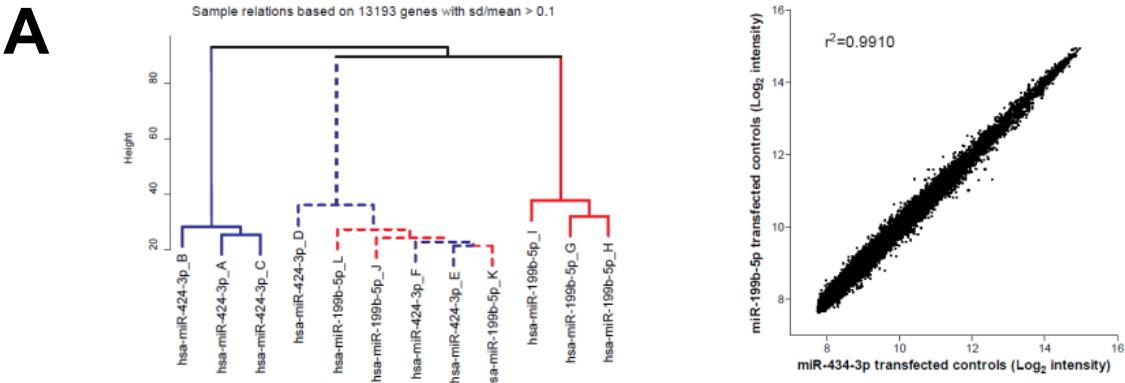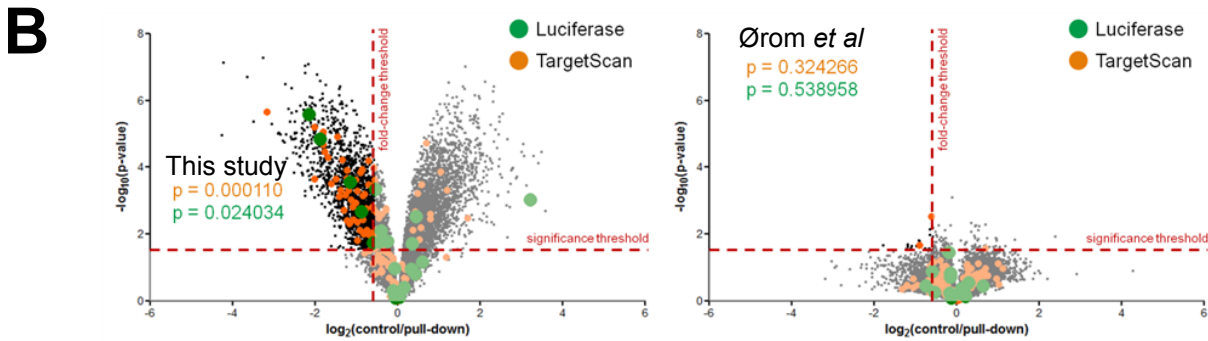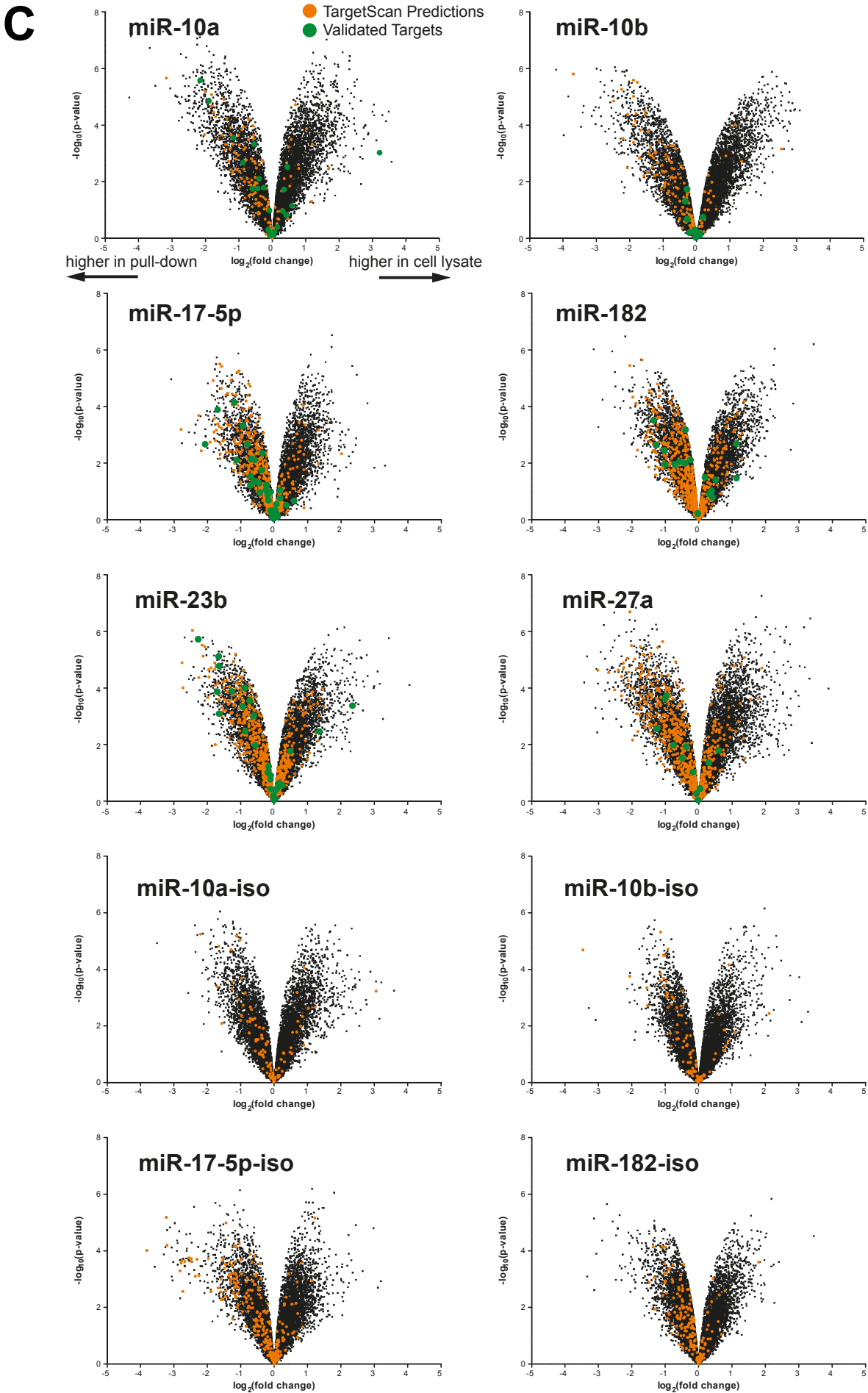

Supplement: Additional file 2: Figure S1 — Biotin pull-downs enrich for predicted and previously validated targets of miRNAs. (A) Left: two different miRNA duplexes, hsa-miR-424-3p (blue) and hsa-miR-199b-5p (red), were transfected into HEK293T cells, independently replicated three times. Total RNA samples (dotted lines) and pull-down miRNA enrichments (solid lines) were assayed by microarray, and clustered using the plotSampleRelations function of lumi. There is a very close relationship between the total RNA samples, even though they have been transfected with two different miRNAs with very different targets. Right: correlation of HEK293T control RNA transfected with either miR-424-3p or miR-199b-5p miRNAs. This demonstrates that there is very little effect of either duplex in this cell line, and that there is no major disruption of the underlying genetic networks upon transfection at this concentration. (B,C) Volcano plots showing the significance of the difference in expression between the indicated pull-down and the mock-transfected control, for all transcripts expressed in control cells. Targets predicted by TargetScan or validated previously via luciferase assay are indicated by orange and green dots, respectively. (B) A comparison between miR-10a biotin pull-down in this study (left) and by Ørom et al.[17] (right). P-values for the enrichment of luciferase validated targets or TargetScan predicted targets are indicated. Red dashed lines indicated the significance threshold and the fold-change threshold used in this study. For the pull-downs performed by Ørom et al., no enrichment of known or predicted targets was observed. (C) Volcano plots for all 10 miRNAs/isomiRs used in this study. [file gb-2014-15-3-r51-S2.pdf]

# SUPPLEMENTARY FIGURE 2

Distribution of log2(fold change)

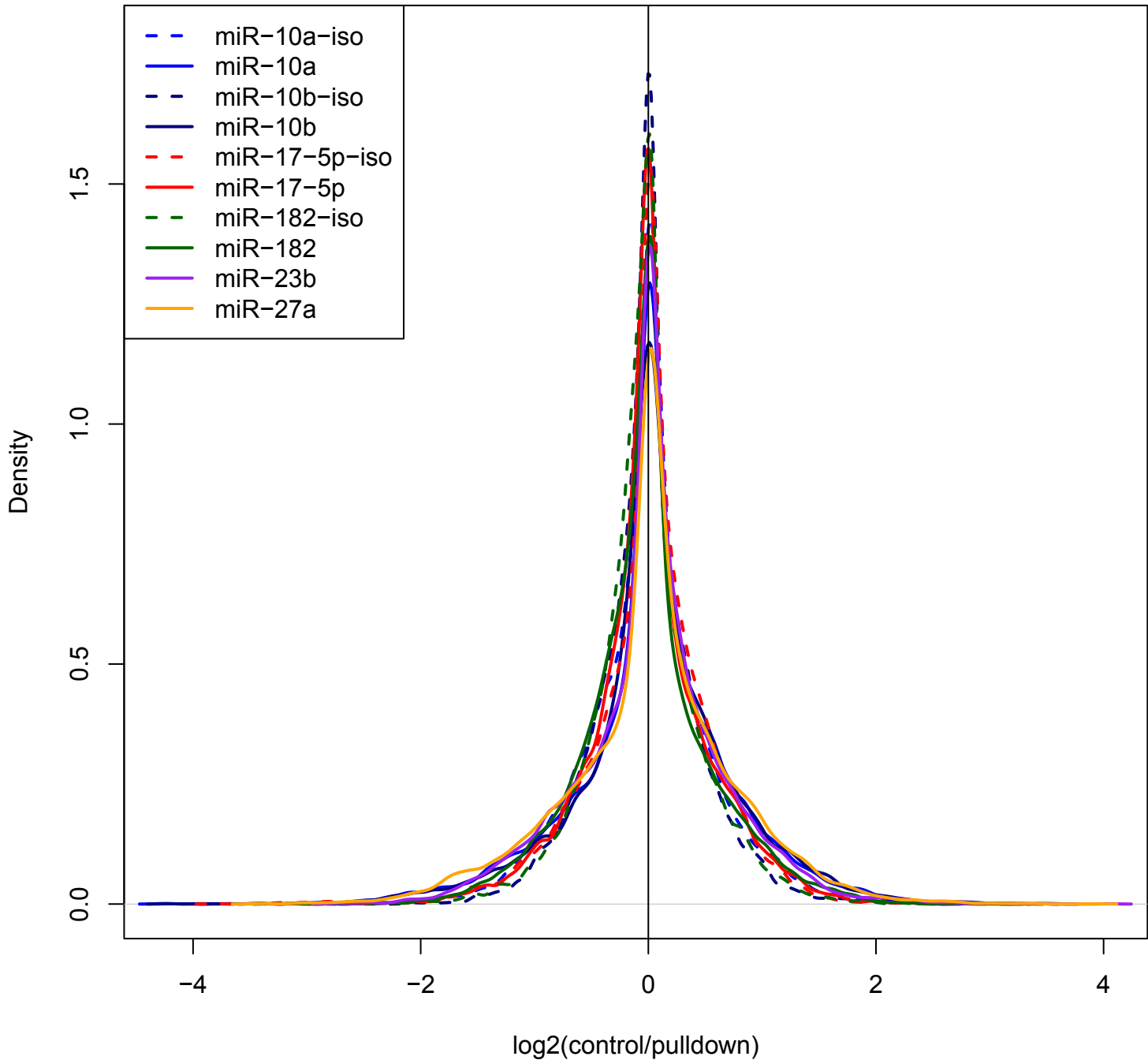

Supplement: Additional file 3: Figure S2 — Distribution of log2 fold-change values for the miRNAs/isomiRs used in this study. Canonical miRNAs are plotted as solid lines. IsomiRs are plotted as dashed lines. [file gb-2014-15-3-r51-S3.pdf]

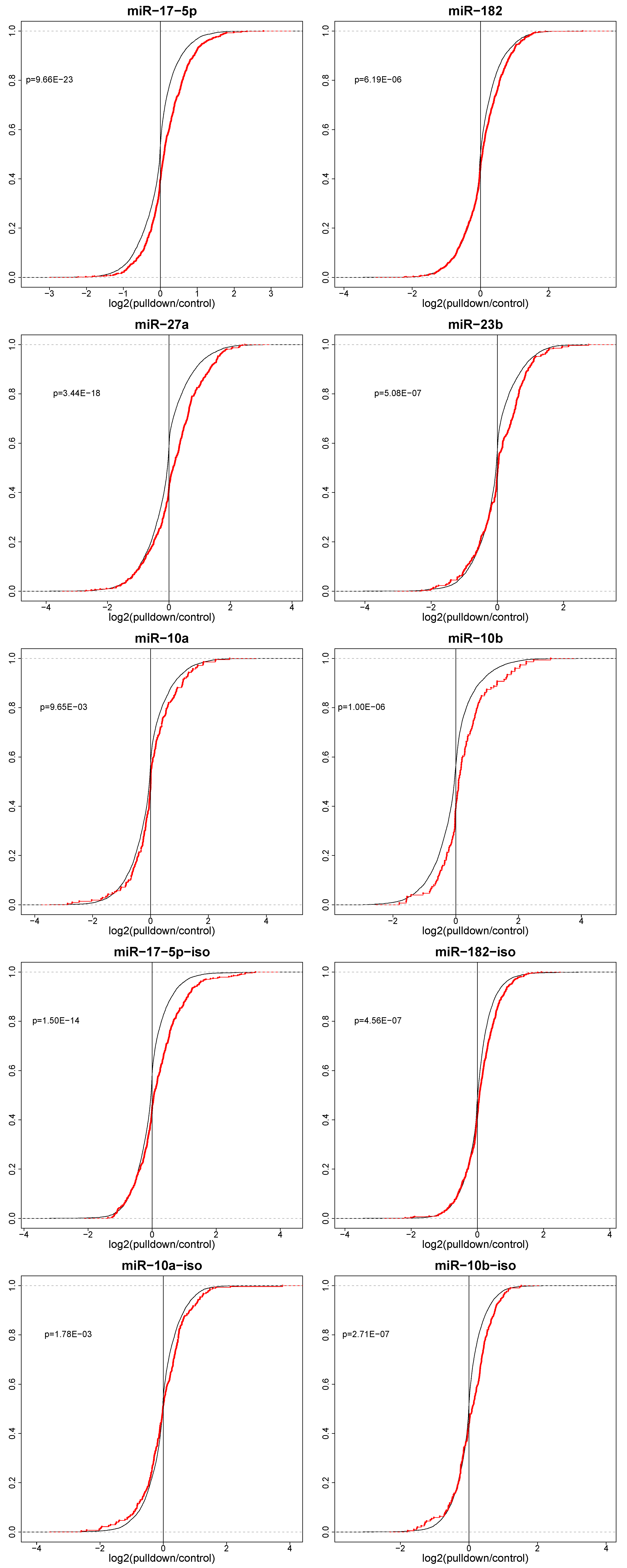

Supplement: Additional file 4: Figure S3 — Targets identified through PAR-CLIP show greater enrichment in the biotin pull-down. Cumulative distribution of log fold-change in the biotin pull-down for transcripts identified as targets via PAR-CLIP [12] or not. Red, canonical transcripts containing at least one CLIP cluster (Table S5 in Additional file 1); black, all other canonical transcripts; p, one-sided P-value from Kolmogorov-Smirnov test for a difference in distributions. [file gb-2014-15-3-r51-S4.png]

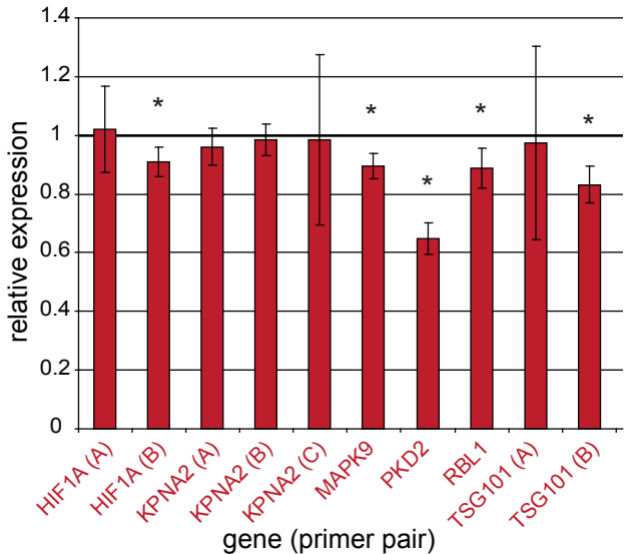

Supplement: Additional file 5: Figure S4 — RT-PCR of genes previously confirmed by luciferase assay. The bar plot indicates the expression of the indicated gene in HEK293T cells transfected with miR-17-5p relative to mock-transfected cells. These genes had previously been shown to be targeted by miR-17-5p in luciferase assays. Error bars indicate 95% confidence intervals calculated over three biological replicates. Asterisks indicate significantly reduced expression compared to mock-transfected cells (one-sided t-test). [file gb-2014-15-3-r51-S5.pdf]

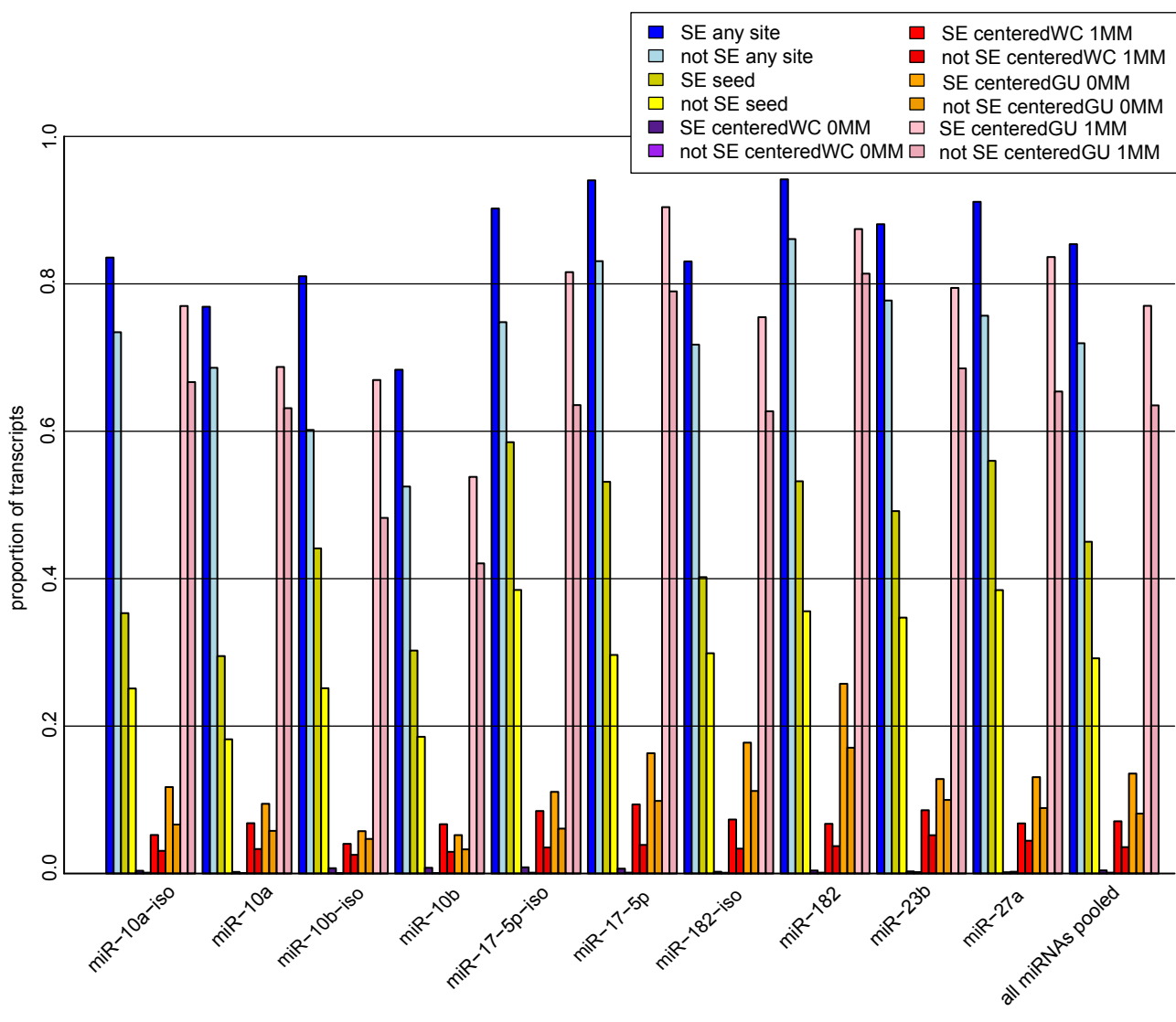

Supplement: Additional file 7: Figure S6 — Proportion of transcripts with miRNA binding sites. Proportion of transcripts with a predicted binding site for the biotinylated miRNA in each pull-down. SE, significantly enriched in pull-down (5% FDR). [file gb-2014-15-3-r51-S7.pdf]

**A**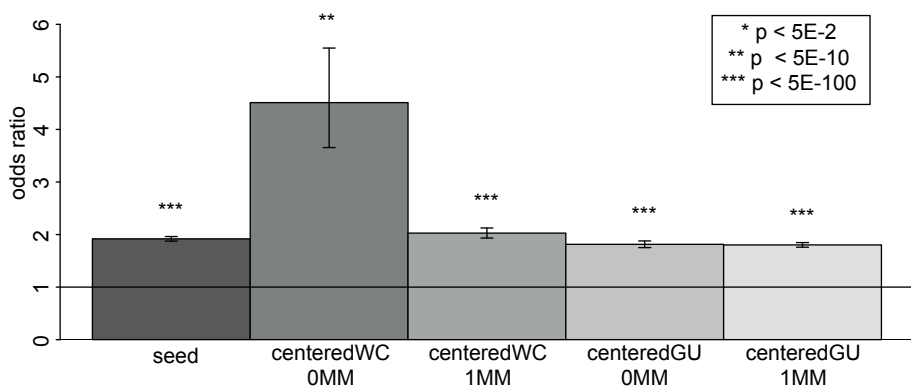**B**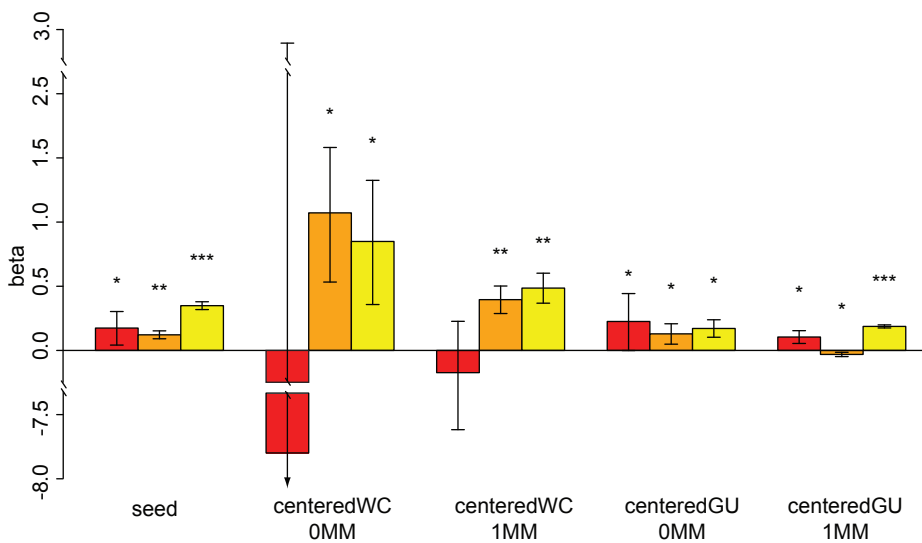**C**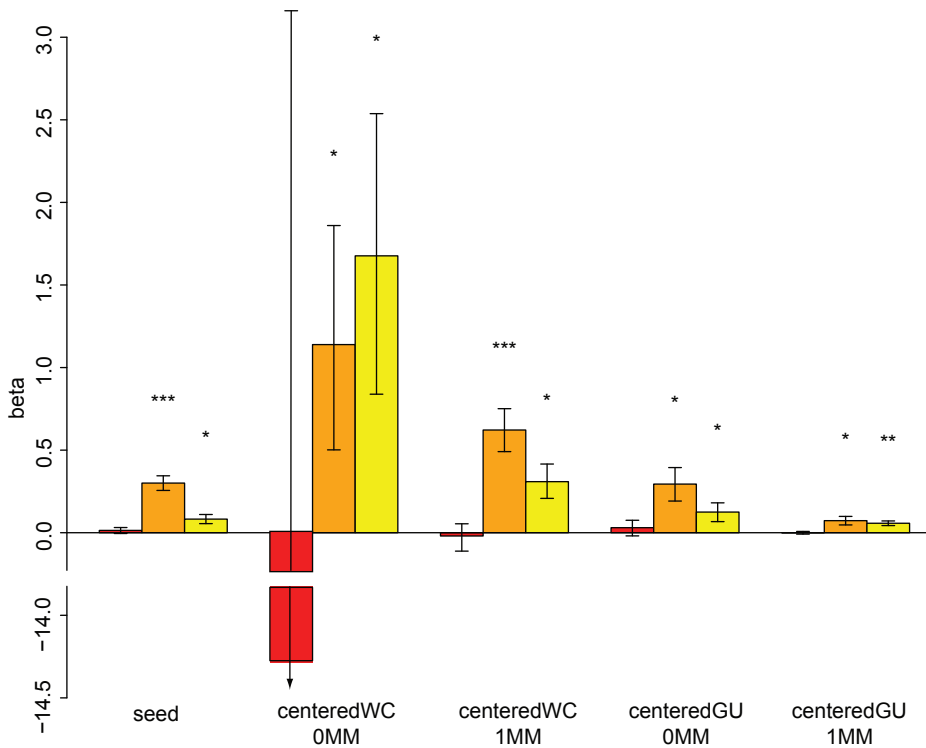

Supplement: Additional file 8: Figure S7 — Effect of site location on enrichment in the biotin pull-down. (A) The odds ratios from Fisher’s exact tests for an enrichment of transcripts with the indicated site type amongst the putative target set including all transcripts. (B,C) Bar plots show the coefficient estimates from a logistic regression of enrichment status on site density (C) or site count (B). Only canonical transcripts were included. Error bars indicate 95% confidence intervals. [file gb-2014-15-3-r51-S8.pdf]

Sample relations based on 14223 genes with sd/mean > 0.1

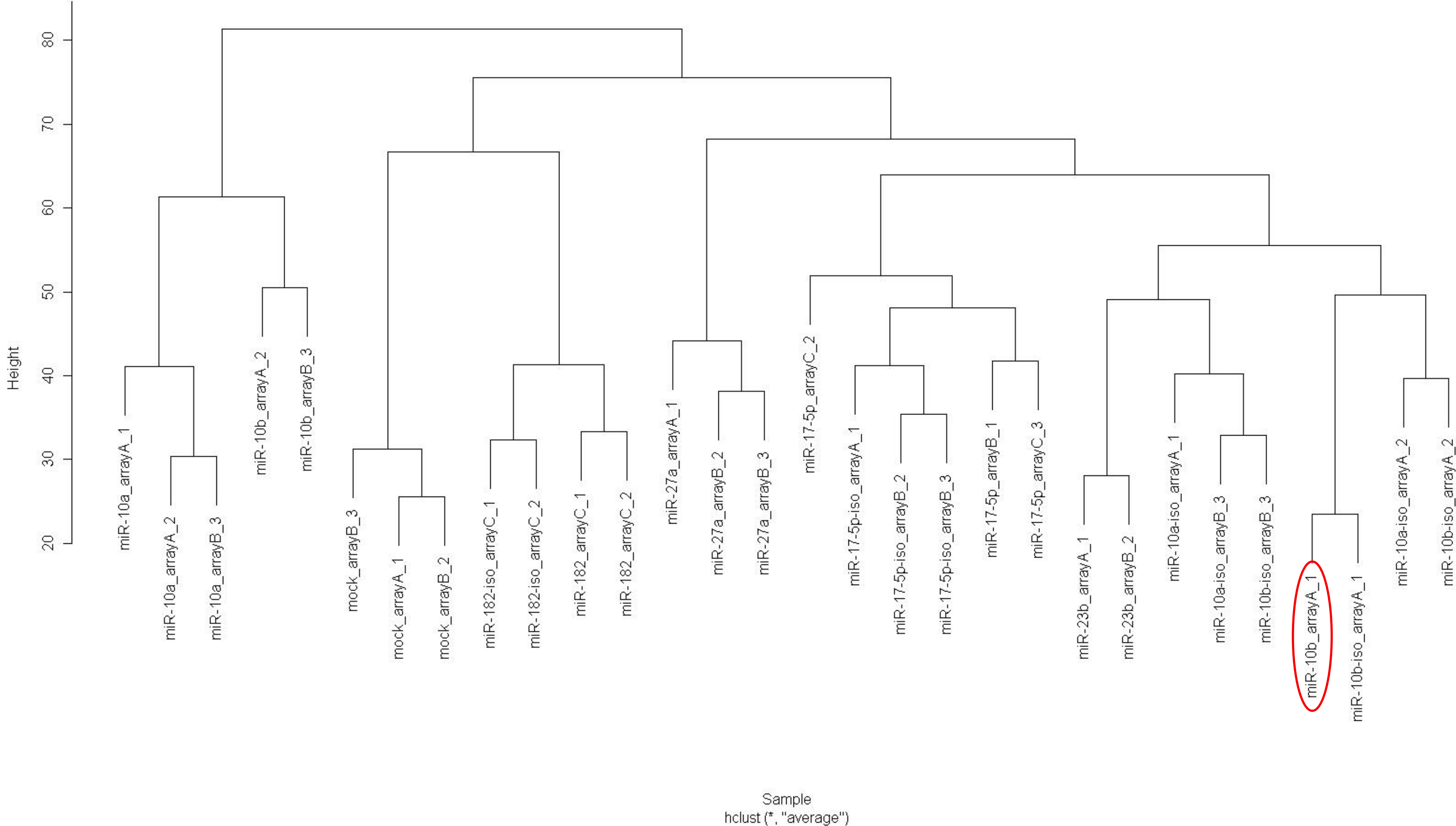

Supplement: Additional file 9: Figure S8 — Hierarchical clustering of microarray data. Clustering was performed using the plotSampleRelations function in the lumi package. Total vertical distance between samples indicates similarity. Arrays A and B were Illumina HT-12 version 4 arrays, and array C was version 3. The miR-10b pull-down sample that clustered on a different branch to the other miR-10a and miR-10b samples (miR-10b_arrayA_1: red circle) was excluded. [file gb-2014-15-3-r51-S9.pdf]
