# Supplementary material for: Imperfect centered miRNA binding sites are common and can mediate repression of target mRNAs
Source: Genome Biol. 2014 Mar 14;15(3):R51. doi: 10.1186/gb-2014-15-3-r51 (PMC4053950; doi:10.1186/gb-2014-15-3-r51)
Supplement: Additional file 6: Figure S5 — Distribution of binding sites in miRNA transcripts. Frequency histograms showing the distribution of the number of each site type per transcript, for each of the 10 miRNAs used in this study (Table 1). [file gb-2014-15-3-r51-S6.pdf]

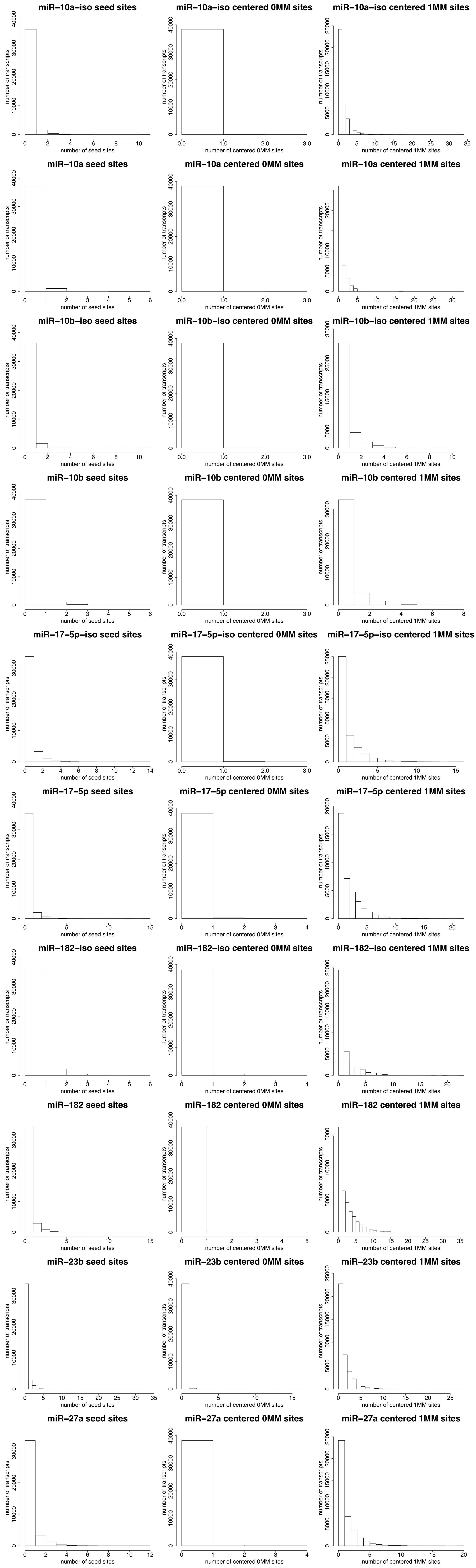
miR−10a−iso seed sites
miR−10a−iso centered 0MM sites
miR−10a−iso centered 1MM sites
miR−10a seed sites
miR−10a centered 0MM sites
miR−10a centered 1MM sites
miR−10b−iso seed sites
miR−10b−iso centered 0MM sites
miR−10b−iso centered 1MM sites
miR−10b seed sites
miR−10b centered 0MM sites
miR−10b centered 1MM sites
miR−17−5p−iso seed sites
miR−17−5p−iso centered 0MM sites
miR−17−5p−iso centered 1MM sites
miR−17−5p seed sites
miR−17−5p centered 0MM sites
miR−17−5p centered 1MM sites
miR−182−iso seed sites
miR−182−iso centered 0MM sites
miR−182−iso centered 1MM sites
miR−182 seed sites
miR−182 centered 0MM sites
miR−182 centered 1MM sites
miR−23b seed sites
miR−23b centered 0MM sites
miR−23b centered 1MM sites
miR−27a seed sites
miR−27a centered 0MM sites
miR−27a centered 1MM sites
number of transcripts
number of seed sites
number of centered 0MM sites
number of centered 1MM sites
